# Sustained dysregulation of iron and glutathione homeostasis induces chronoferroptosis, a persistent ferroptotic adaptation in neuronal cells

**DOI:** 10.1038/s41420-026-03208-6

**Published:** 2026-06-18

**Authors:** Nawab John Dar, David Soriano-Castell, Pamela Maher

**Affiliations:** Department of Cellular Neurobiology, The Salk Institute for Biological Studies, La Jolla, CA USA

**Keywords:** Cellular neuroscience, Stress and resilience

## Abstract

Although iron accumulates in brain regions impacted by neurodegenerative diseases such as Alzheimer’s and Parkinson’s, how chronic elevated iron levels contribute to neuronal dysfunction remains unclear. Here, we show that sustained iron overload, but not acute exposure, leads to a state of ferroptotic stress where nerve cells remain viable but become hypersensitive to oxidative injury. Retinoic acid-differentiated SH-SY5Y neuronal cells were exposed to acute (6–8 h) or chronic (9 days) iron loading to model transient versus prolonged age-related iron stress. While acute iron exposure produced minimal biochemical changes and did not sensitize cells to oxidative or ferroptotic challenges, chronic iron exposure induced ferritin upregulation, mitochondrial superoxide accumulation, suppression of GPX4 expression, elevated lipid peroxidation and loss of cellular glutathione (GSH). In addition, chronic but not acute GSH depletion by buthionine sulfoximine (BSO) recapitulated the iron-induced phenotype. Cells under chronic ferroptotic stress exhibited increased sensitivity not only to the ferroptosis inducer RSL-3 but also to hydrogen peroxide. Ferrostatin-1 significantly mitigated these effects suggesting that lipid peroxidation drives this state. Together, these findings demonstrate that, in contrast with acute exposure, chronic disruption of iron homeostasis with consequent GSH depletion remodels cellular redox homeostasis over time, inducing a state we term chronoferroptosis: a persistent ferroptotic adaptation characterized by coordinated alterations in iron-handling and antioxidant defense proteins that may represent early vulnerability to neurodegenerative pathology. Thus, these studies highlight the importance of sustained stress paradigms for modeling the progressive nature of neurodegenerative diseases.

**Figure:**
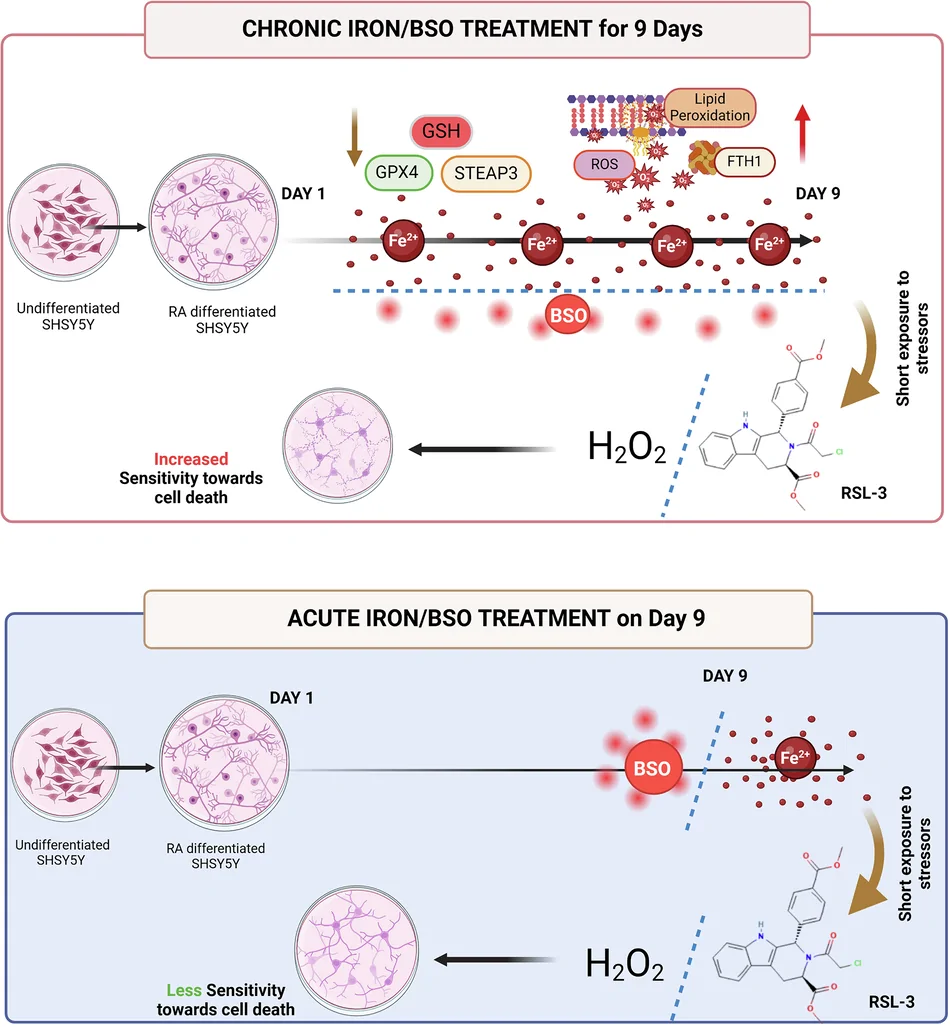
Graphical abstract illustrating how prolonged iron or BSO exposure drives a persistent ferroptotic stress state in RA differentiated SH-SY5Y cells. These cells were subjected to either acute exposure for 6–8 h or chronic exposure for 9 days. Acute iron or BSO treatment caused minimal biochemical changes and did not increase vulnerability to oxidative or ferroptotic challenges. In contrast, chronic exposure stimulated ferritin production, mitochondrial superoxide buildup, GSH depletion, reductions in GPX4 expression and increased lipid peroxidation without altering cell viability. However, these chronically stressed neuronal cells became markedly more sensitive to secondary stressors such as RSL-3 or hydrogen peroxide resulting in decreases in cell viability, whereas viability was not altered in acutely treated cells.

Graphical abstract illustrating how prolonged iron or BSO exposure drives a persistent ferroptotic stress state in RA differentiated SH-SY5Y cells. These cells were subjected to either acute exposure for 6–8 h or chronic exposure for 9 days. Acute iron or BSO treatment caused minimal biochemical changes and did not increase vulnerability to oxidative or ferroptotic challenges. In contrast, chronic exposure stimulated ferritin production, mitochondrial superoxide buildup, GSH depletion, reductions in GPX4 expression and increased lipid peroxidation without altering cell viability. However, these chronically stressed neuronal cells became markedly more sensitive to secondary stressors such as RSL-3 or hydrogen peroxide resulting in decreases in cell viability, whereas viability was not altered in acutely treated cells.

## Highlights

- Chronic, but not acute, iron or GSH imbalance induces chronoferroptosis in neuronal cells.
- Sustained increases in iron or decreases in GSH suppress GPX4 levels thereby compromising cellular antioxidant defenses.
- Mitochondrial ROS and lipid peroxidation increase under chronic stress without immediate cell death.
- Chronoferroptosis increases cellular vulnerability to additional oxidative stress.
- Ferrostatin-1 reduces ferroptotic vulnerability suggesting an important role for lipid peroxidation in this process.

## Introduction

Iron accumulation is consistently observed in neurodegenerative disorders and, especially, in Alzheimer’s disease (AD) and Parkinson’s disease (PD) [1–5]. Post-mortem analyses reveal substantial iron enrichment in the substantia nigra of PD patients and in the hippocampus of those with AD, where iron burden correlates with neuronal loss and cognitive decline [6–9]. Imaging studies further demonstrate that increased iron levels predict accelerated neurodegeneration, suggesting it as both a marker and a mediator of disease progression [9–12]. Parallel to this, early depletion of GSH, the major intracellular antioxidant, has been detected in vulnerable regions before the onset of evident pathology, particularly within the substantia nigra in PD [13–15]. This convergence of iron dysregulation and GSH depletion forms the mechanistic core of a specific, regulated cell death pathway called oxytosis/ferroptosis (hereafter ferroptosis) which is distinct from apoptosis and necrosis [16]. This pathway was originally characterized in nerve cells by our laboratory and named oxytosis, a process initiated by inhibition of the cystine/glutamate antiporter, leading to GSH depletion, accumulation of reactive oxygen species, and lethal lipid peroxidation [17, 18]. This same iron-dependent oxidative death cascade, driven by the failure of glutathione peroxidase 4 (GPX4) to neutralize lipid hydroperoxides, was later termed ferroptosis [19]. Both terms describe an identical pathway [20]. Thus, ferroptosis represents a mechanistic link between the pathological hallmarks of neurodegeneration, namely iron accumulation and GSH loss, and the selective neuronal death observed in affected regions in neurodegenerative diseases.

The regulation and execution of ferroptosis depends upon a coordinated network of redox and iron-handling proteins that together maintain neuronal survival under oxidative pressure [21]. Iron metabolism is, in part, regulated by ferritin heavy chain (FTH1), which converts Fe²⁺ to Fe³⁺ and stores it in a redox-inactive form, and by STEAP-3, an endosomal ferrireductase that converts Fe³⁺ to Fe²⁺ to sustain the labile iron pool [22, 23]. Perturbations in iron homeostasis increase the availability of redox-active iron, promoting lipid peroxidation via Fenton chemistry [24]. Iron-induced oxidative stress can also deplete cellular GSH, creating a secondary impairment of antioxidant defenses. GSH serves as the essential cofactor for GSH peroxidase 4 (GPX4), the central defense enzyme that reduces lipid hydroperoxides to alcohols [25–27]. Loss or inhibition of GPX4 in neurons leads to rapid hippocampal and cortical degeneration with extensive lipid oxidation [28], while selective GPX4 deficiency in dopaminergic neurons accelerates motor dysfunction, linking ferroptosis vulnerability to PD-relevant pathology [29]. The catalytic activity of GPX4 requires sustained GSH availability which is maintained by the cystine/glutamate antiporter system xc⁻ via its specific subunit xCT (SLC7A11) that imports cystine in exchange for glutamate, providing cysteine for GSH synthesis [30]. Pharmacological depletion of GSH similarly sensitizes neurons to ferroptosis [31], demonstrating that GSH loss, whether from oxidative consumption or impaired synthesis, compromises the GSH-GPX4 antioxidant axis.

While these mechanisms are well established under acute conditions, neurodegenerative disorders develop in a fundamentally different temporal context. Most mechanistic insights into ferroptosis arise from acute genetic or pharmacological manipulations that trigger rapid cell death within hours. These models capture the terminal execution phase but do not reflect the chronic oxidative environment that neurons experience in most neurodegenerative diseases, where iron accumulation and GSH depletion occur over much longer periods of time. However, we have suggested that persistent, low-level iron accumulation and gradual GSH loss can coexist for extended periods [18] suggesting the possibility of a sustained sub-lethal state. This observation led us to hypothesize a phenomenon we term chronoferroptosis (chronic ferroptotic stress) which is a persistent ferroptotic adaptation distinct from acute ferroptotic cell death [32–34]. We define chronoferroptosis as a stable, non-lethal cellular state in which neurons survive under sustained oxidative pressure but exhibit coordinated alterations in key ferroptosis-regulatory proteins (GPX4, xCT, STEAP-3, ferritin) that shift cells toward increased ferroptotic susceptibility [18, 20, 34]. Unlike acute ferroptosis, which rapidly progresses to cell death within hours, chronoferroptosis represents an adaptive-yet-vulnerable intermediate state that can persist without immediate lethality [35, 36]. Importantly, this intermediate state cannot be captured by acute exposure paradigms, necessitating chronic stress models that better reflect the temporal progression of neurodegenerative diseases.

To address this gap, the present study employs a chronic exposure paradigm using retinoic acid (RA)-differentiated SH-SY5Y cells to model sustained iron overload and GSH depletion over nine days. This cell model has been extensively validated for studying oxidative stress mechanisms and ferroptotic cell death pathways relevant to neurodegeneration [37]. Our experimental design employs short-term (6–8 h, acute) and long-term (9 day, chronic) cell treatments to recapitulate transient versus sustained stress. Our findings reveal that both chronic iron exposure and GSH depletion drive coordinated remodeling of cellular redox homeostasis that dramatically increases vulnerability to additional oxidative challenges, establishing the chronoferroptosis phenotype [34]. Thus, this model provides new mechanistic insight into how sustained alterations in iron and GSH reprograms neuronal redox homeostasis and primes neuronal cells for degeneration, bridging the gap between acute ferroptotic triggers and the slow progression of neurodegenerative diseases.

## Results

### Chronic iron accumulation and GSH depletion remodel labile iron pools and induce ferritin expression

**Fig. 1:**
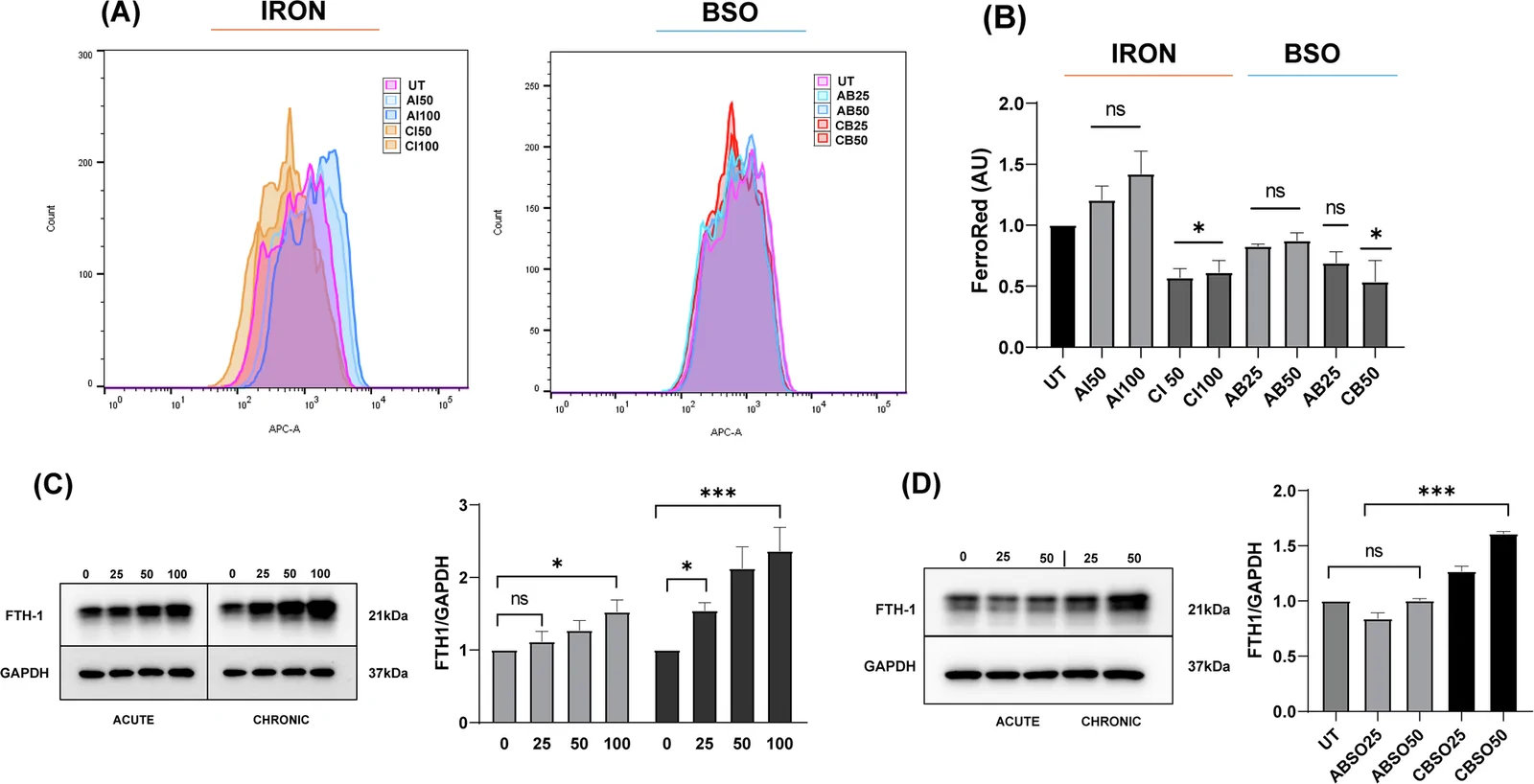
Chronic iron accumulation and GSH depletion alter labile iron pools and induce ferritin expression in differentiated SH-SY5Y cells. **A** Representative FerroRed flow-cytometry histograms showing intracellular labile Fe²⁺ following treatment with increasing concentrations of FeSO₄ or BSO under acute (6–8 h) and chronic (9-day) paradigms. Acute iron exposure produced a rightward fluorescence shift consistent with a transient increase in labile Fe²⁺, whereas chronic exposure resulted in a leftward shift indicating a reduction in the measurable labile Fe²⁺ pool. **B** Quantification of FerroRed median fluorescence intensity (MFI; arbitrary units, AU) normalized to untreated controls (UT). Acute iron exposure showed a dose-dependent increase in labile Fe²⁺, reaching significance at 100 µM (*p < 0.05), whereas chronic exposure produced a significant reduction at 50 µM and 100 µM (*p < 0.05). For BSO, acute treatment did not significantly change the signal, while chronic 50 µM BSO exposure resulted in a modest but significant decrease (*p < 0.05). Bars represent mean ± SEM (*n* = 3). **C** Representative immunoblots for ferritin heavy chain (FTH1) and GAPDH following acute and chronic iron exposure, with densitometric quantification of FTH1 normalized to GAPDH. Acute 100 µM iron caused a mild but significant increase in FTH1 (*p < 0.05), whereas chronic iron treatment induced robust upregulation at all doses (25 µM, *p < 0.05; 50–100 µM, ***p < 0.001). **D** Representative immunoblots for FTH1 and GAPDH following acute and chronic BSO exposure, with densitometric quantification of FTH1 normalized to GAPDH. Chronic BSO exposure significantly increased FTH1 expression at both 25 µM and 50 µM (***p < 0.001) while the effect of acute BSO treatment was not significant. Color codes for flow cytometry histograms in panel (**A**) are as follows. Iron histogram: UT, magenta; AI50, blue; AI100, blue; CI50, orange; CI100, orange. BSO histogram: UT, magenta; AB25, cyan/blue; AB50, blue; CB25, red; CB50, red. UT untreated, AI acute iron, CI chronic iron, AB acute BSO, CB chronic BSO, MFI median fluorescence intensity, AU arbitrary units, ns not significant.

Our experimental design employed two complementary stress paradigms: (1) chronic iron accumulation to model age-related brain iron overload and (2) chronic GSH depletion using buthionine sulfoximine (BSO) to investigate the role of GSH loss independent of exogenous iron, since preliminary observations suggested a mechanistic link between iron and GSH dysregulation. To test our hypothesis that long-term dyshomeostasis of iron might drive chronic ferroptotic stress in a significantly different manner than acute disruptions, we first investigated how the temporal dimension of iron exposure alters neuronal iron handling. RA-differentiated SH-SY5Y cells were exposed to ferrous sulfate (FeSO₄, 50–100 µM) or BSO (25–50 µM) under acute (6–8 h) and chronic (9-day) paradigms, enabling direct comparison of short-term versus sustained stress responses. Flow-cytometric analysis with FerroRed (Fig. 1A, B) revealed a notable temporal difference in iron handling. Acute iron exposure produced a rightward fluorescence shift, consistent with a transiently increased labile Fe²⁺ pool (LIP), reaching significance at 100 µM (*p < 0.05). This transient elevation demonstrates that short-term iron exposure briefly increases the LIP, possibly without triggering adaptive responses. In contrast, chronic exposure resulted in a significant reduction in the FerroRed signal at both 50 µM and 100 µM (*p < 0.05), suggesting sequestration of Fe²⁺ into storage forms or increased export. This divergent response of increased labile iron under acute conditions versus decreased labile iron under chronic conditions demonstrates fundamentally different cellular adaptations to transient versus sustained iron exposure.

These results suggest that prolonged iron exposure initiates alterations in iron homeostasis that reduce the measurable labile Fe²⁺ pool which could represent an adaptive response that would be absent during acute treatment. To assess whether this reduction reflects increases in ferritin-mediated storage, we evaluated ferritin heavy chain (FTH1) expression under both temporal paradigms (Fig. 1C). Indeed, the results showed a strong temporal contrast. Acute 100 µM iron exposure modestly increased FTH1 levels (*p < 0.05), whereas chronic iron treatment induced robust ferritin upregulation across all concentrations (25 µM, *p < 0.05; 50–100 µM, ***p < 0.001). This represents a significantly different response under chronic versus acute conditions which could indicate that sustained iron exposure triggers a coordinated adaptive iron-storage program.

BSO treatment followed a similar temporal pattern. Acute exposure did not significantly affect labile iron, whereas chronic 50 µM BSO led to a modest but significant decrease (*p < 0.05) (Fig. 1A, B). Chronic BSO exposure also significantly elevated FTH1 expression at both 25 µM and 50 µM (***p < 0.001), while acute treatment did not induce significant differences (Fig. 1D). The inverse relationship in the chronic state between FerroRed fluorescence and FTH1 upregulation suggests that most redox-active Fe²⁺ becomes stored within ferritin during chronic ferroptotic stress, reducing its cytosolic availability. Together, these findings are consistent with the idea that sustained iron overload or GSH depletion triggers an adaptive iron-storage response that limits labile Fe²⁺-a coordinated cellular remodeling that is minimal or absent during acute exposure.

### Chronic iron and GSH depletion suppress antioxidant and iron-handling proteins compared to acute treatments

**Fig. 2:**
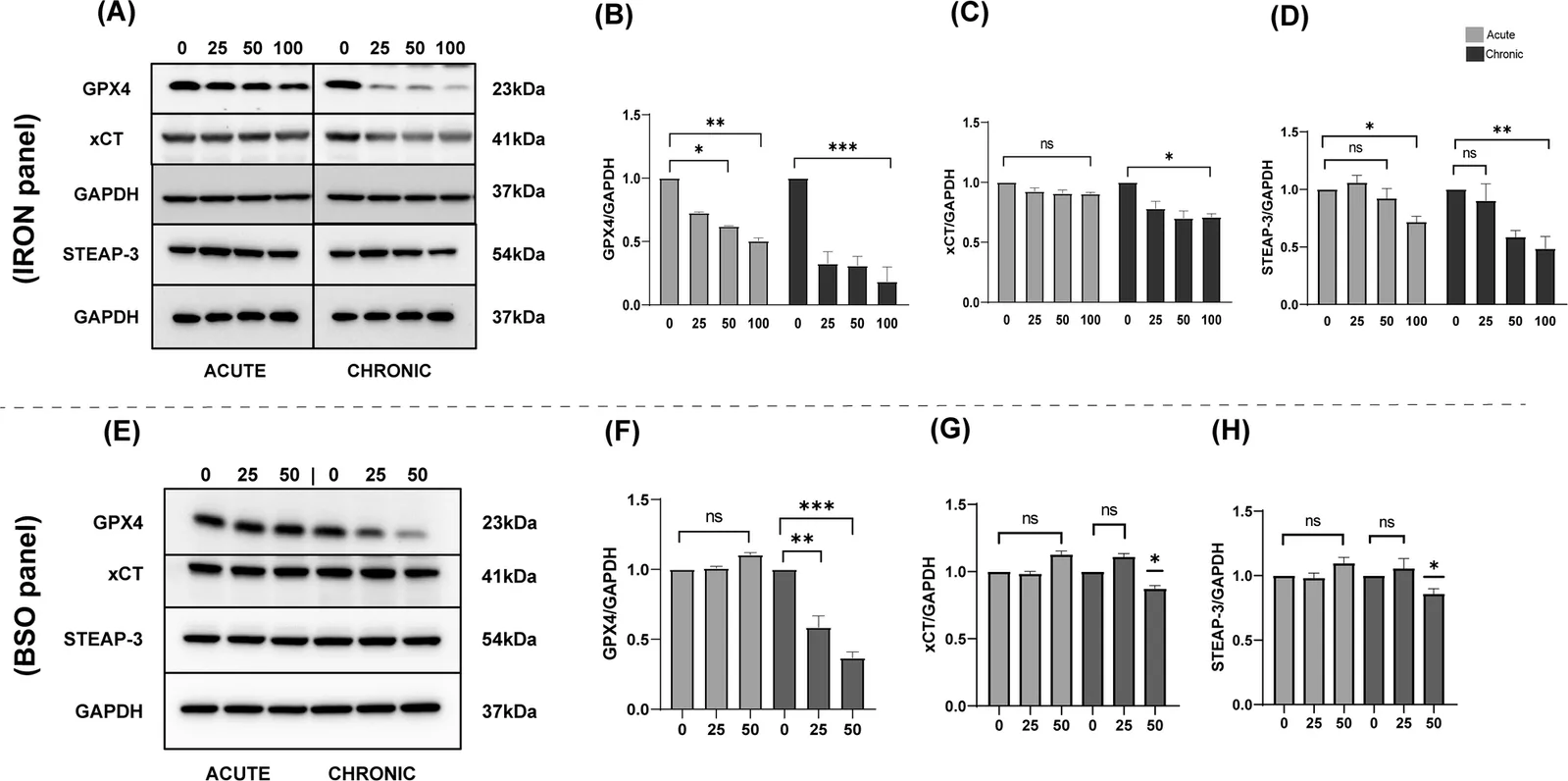
Chronic iron accumulation and GSH depletion suppress antioxidant and iron-handling proteins. **A**, **E** Representative immunoblots showing GPX4, xCT (SLC7A11), and STEAP-3 expression following acute (6–8 h) and chronic (9-day) exposure to FeSO₄ or BSO in RA-differentiated SH-SY5Y cells. **B**–**D** Densitometric quantification of GPX4, xCT, and STEAP-3 protein levels normalized to GAPDH in iron-treated cultures. Chronic iron exposure markedly reduced GPX4 levels (***p < 0.001) relative to acute treatment while xCT showed a modest but significant decrease specifically under chronic conditions (*p < 0.05). For STEAP-3, acute iron exposure produced a significant reduction at 100 µM (*p < 0.05), whereas chronic iron exposure produced significant reductions at 50 µM and 100 µM (**p < 0.01), with chronic 25 µM showing no significant change. **F**–**H** Quantification of GPX4, xCT, and STEAP-3 normalized to GAPDH in BSO-treated cultures. Chronic BSO exposure significantly reduced GPX4 at both 25 µM (**p < 0.01) and 50 µM (***p < 0.001) with concurrent decreases in xCT and STEAP-3 at 50 µM (*p < 0.05). Acute BSO exposure produced no significant changes in these proteins. Bars represent mean ± SEM (*n* = 3).

Because ferroptosis is governed by both redox and iron-regulatory networks [38] we next examined whether the temporal dimension of iron exposure differentially affects GPX4, xCT (SLC7A11) and STEAP-3 expression. These proteins represent critical nodes in the ferroptosis defense machinery and their regulation under acute versus chronic stress could determine cellular vulnerability. As shown in Fig. 2A–D, chronic iron exposure markedly reduced GPX4 (***p < 0.001) and STEAP-3 protein levels compared with acute treatment. Acute iron exposure produced a significant reduction in STEAP-3 at 100 µM (*p < 0.05), whereas chronic iron exposure produced significant reductions at 50 µM and 100 µM (**p < 0.01), with chronic 25 µM showing no significant change. In the context of chronic iron loading, STEAP-3 downregulation may represent an adaptive response to limit further Fe²⁺ release from endosomal stores, thereby restricting substrate availability for iron-catalyzed oxidative reactions. Meanwhile, GPX4 and xCT also showed significant reductions (*p < 0.05) specifically under chronic conditions. While STEAP-3 downregulation could be protective by limiting Fe²⁺ mobilization, the concurrent suppression of GPX4 and xCT under chronic but not acute iron exposure indicates a time-dependent erosion of antioxidant capacity. Given that chronic, but not acute, iron exposure depletes GPX4 and xCT, we hypothesized that direct GSH depletion might produce similar molecular changes independent of iron supplementation. To test this, we examined the same proteins in BSO-treated cultures (Fig. 2E–H). Chronic BSO exposure caused a significant reduction in GPX4 at both 25 µM (**p < 0.01) and 50 µM (***p < 0.001) with concurrent decreases in xCT and STEAP-3 only at 50 µM (*p < 0.05). This shared temporal signature, minimal changes under acute exposure but coordinated suppression under chronic treatment, indicates that GSH depletion also produces time-dependent alterations in ferroptosis-regulatory proteins. Taken together, these results highlight the fundamental differences between short-term and sustained iron exposure and GSH depletion, showing similar mechanistic patterns consistent with a common signature for chronic ferroptotic stress.

### Chronic iron accumulation and GSH depletion reduce total GSH levels, elevate mitochondrial superoxide, and alter mitochondrial membrane potential

**Fig. 3:**
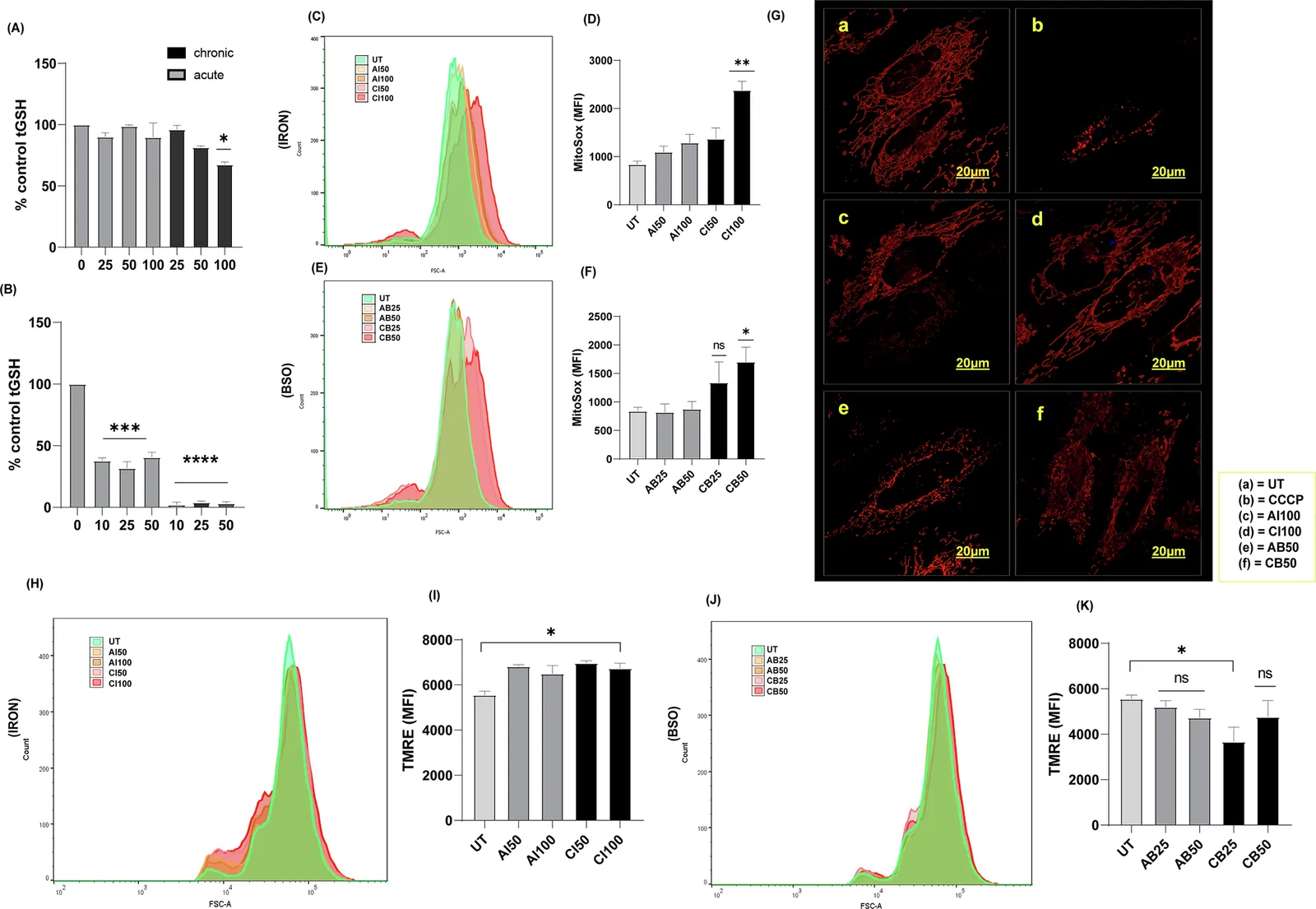
Chronic iron accumulation and GSH depletion reduce total GSH levels, elevate mitochondrial superoxide, and alter mitochondrial membrane potential. **A** Total intracellular GSH (tGSH) levels following iron treatment expressed as a percentage of untreated controls. Acute iron (25–100 µM, 8 h) and chronic 50 µM iron do not significantly alter tGSH whereas chronic 100 µM iron causes a modest but significant reduction (*p < 0.05). **B** Total GSH levels following BSO (10, 25, 50 µM). Acute BSO (10–50 µM) causes a significant but incomplete reduction in GSH (approximately 50% decrease, ***p < 0.001) whereas chronic BSO (9 days) results in near-complete GSH depletion across all concentrations tested (10–50 µM, ****p < 0.0001). **C** Representative flow-cytometry histograms of MitoSOX Red fluorescence showing mitochondrial reactive oxygen species (ROS) accumulation following acute and chronic iron treatments (50 and 100 µM). **D** Quantification of MitoSOX MFI normalized to untreated controls shows a pronounced increase in mitochondrial ROS under chronic iron exposure, most prominently at 100 µM (**p < 0.01), whereas acute exposure induces minimal changes. **E** Representative MitoSOX histograms following acute and chronic BSO treatments (25 and 50 µM). **F** Quantification demonstrating that chronic BSO exposure significantly increases mitochondrial ROS compared with acute treatment with 50 µM BSO reaching significance (*p < 0.05). Bars represent mean ± SEM (*n* = 3). **G** Representative high-magnification live-cell Airyscan confocal images (63× oil immersion objective, scale bar = 20 µm) of TMRE-stained RA-differentiated SH-SY5Y cells under six conditions: (a) untreated (UT), (b) CCCP (20 µM, positive control for mitochondrial depolarization), (c) acute iron 100 µM (AI100), (d) chronic iron 100 µM (CI100), (e) acute BSO 50 µM (AB50), and (f) chronic BSO 50 µM (CB50), as identified by the panel key shown in the lower right of the figure. TMRE fluorescence is shown in red. Untreated cells display a well-defined, elongated mitochondrial network with strong and uniform TMRE signal. CCCP-treated cells show a marked reduction in TMRE signal, confirming assay sensitivity and directionality. CI100 cells show visibly stronger TMRE fluorescence relative to untreated and AI100 cells, consistent with elevated mitochondrial membrane potential under chronic iron loading. CB50 cells show a visibly reduced TMRE signal relative to untreated controls, consistent with partial mitochondrial depolarization under sustained GSH depletion. **H** Representative TMRE flow cytometry histograms showing mitochondrial membrane potential following acute (AI50, AI100) and chronic (CI50, CI100) iron treatments relative to untreated controls (UT). **I** Quantification of TMRE MFI normalized to untreated controls shows elevated mitochondrial membrane potential across all iron-exposed groups, with the 100 µM chronic iron-treated group reaching statistical significance (*p < 0.05). Bars represent mean ± SEM (*n* = 3). **J** Representative TMRE flow cytometry histograms showing mitochondrial membrane potential following acute (AB25, AB50) and chronic (CB25, CB50) BSO treatments relative to untreated controls (UT). **K** Quantification of TMRE MFI normalized to untreated controls. Acute BSO at both doses did not significantly alter TMRE MFI (ns). Chronic BSO at 25 µM (CB25) produced a statistically significant reduction in TMRE fluorescence (*p < 0.05), while chronic BSO at 50 µM (CB50) showed a consistent downward trend that did not reach statistical significance (ns). Bars represent mean ± SEM (*n* = 3). Color codes for all flow cytometry histograms in this figure (panels **C**, **E**, **H**, and **J**) are as follows: UT, green; lower acute dose (AI50 or AB25), light orange; higher acute dose (AI100 or AB50), orange; lower chronic dose (CI50 or CB25), light red; higher chronic dose (CI100 or CB50), dark red. The same color assignment is maintained across all four histograms. UT untreated, AI acute iron, CI chronic iron, AB acute BSO, CB chronic BSO, MFI median fluorescence intensity, TMRE tetramethylrhodamine ethyl ester, ROS reactive oxygen species, CCCP carbonyl cyanide 3-chlorophenylhydrazone, ns not significant.

Given that GPX4 requires GSH as a cofactor, we next measured total intracellular GSH (tGSH) to determine whether chronic iron or BSO exposure compromises GSH homeostasis in a time-dependent manner (Fig. 3A, B). The temporal pattern for iron treatment was clear: neither acute iron treatment (8 h at 25–100 µM) nor chronic exposure at lower concentrations (50 µM) significantly altered GSH levels. However, chronic exposure to 100 µM iron caused a modest but significant reduction in GSH (*p < 0.05) compared to untreated controls. Importantly, acute exposure to the same 100 µM iron concentration did not alter GSH levels, demonstrating that GSH loss requires sustained, not transient, iron exposure (Fig. 3A). The observation that chronic but not acute iron exposure reduces GSH levels prompted us to investigate whether the temporal dimension of direct GSH depletion produces similar effects. As expected, BSO treatment produced time-dependent GSH depletion (Fig. 3B). Acute BSO (8 h at 10–50 µM) demonstrated a significant but incomplete reduction in GSH (~50% decrease, ***p < 0.001) whereas chronic BSO exposure (9 days) resulted in near-complete depletion of measurable GSH across all concentrations (10–50 µM, ****p < 0.0001). The temporal difference, where there is partial GSH reduction under acute conditions versus almost complete GSH loss under chronic conditions, demonstrates that even with direct biosynthetic blockade, the duration of GSH depletion determines the severity of redox dysfunction.

Since mitochondria are major sources of reactive oxygen species (ROS) during ferroptosis [39] and GSH is critical for maintaining mitochondrial redox homeostasis [40], we next assessed whether chronic iron or GSH depletion enhances mitochondrial superoxide production. Mitochondrial superoxide generation was measured using MitoSOX Red (Fig. 3C–F). The temporal difference was pronounced: chronic iron exposure significantly increased mitochondrial ROS, most prominently at 100 µM (**p < 0.01), whereas acute exposure caused minimal change (Fig. 3C, D).

We next investigated whether direct GSH depletion by BSO produces similar effects on mitochondria. Acute BSO exposure did not elevate mitochondrial ROS while chronic BSO exposure produced significantly greater mitochondrial ROS elevation relative to acute treatment, with 50 µM BSO reaching significance (*p < 0.05) (Fig. 3E, F). This parallels the temporal pattern observed with iron treatment. These findings demonstrate distinct yet convergent temporal mechanisms: chronic iron raises oxidative stress over time and steadily reduces GSH, while chronic BSO blocks GSH synthesis and drives its complete loss. Even though these treatments act through different routes, both eventually push cells into a GSH-deficient state with high mitochondrial ROS. The absence of this shift in short-term treated cells highlights the importance of sustained stress to model the chronic, sub-toxic ferroptotic stress that could occur in vivo.

To determine whether these redox changes were accompanied by functional alterations in mitochondrial membrane potential, ΔΨm was assessed using TMRE by both Airyscan confocal microscopy and quantitative flow cytometry, with carbonyl cyanide 3-chlorophenylhydrazone (CCCP, 20 μM) included as a positive control for mitochondrial membrane depolarization (Fig. 3G–K). Representative high-magnification Airyscan images (63× oil immersion objective, Fig. 3G) revealed qualitatively distinct mitochondrial responses across conditions. Untreated cells displayed an organized, elongated mitochondrial network with strong and uniform TMRE fluorescence, consistent with intact membrane potential. CCCP-treated cells showed a marked reduction in TMRE signal and visibly altered mitochondrial morphology, confirming the sensitivity and directionality of the assay. Acute iron-treated cells (AI100) displayed a TMRE staining pattern broadly similar to untreated controls, whereas chronic iron-treated cells (CI100) showed visibly stronger TMRE fluorescence relative to both untreated and acute iron-treated cells, indicating an elevated mitochondrial membrane potential under sustained iron loading. In contrast, acute BSO-treated cells (AB50) were largely indistinguishable from untreated controls, while chronic BSO-treated cells (CB50) showed a visibly reduced TMRE signal relative to untreated controls, consistent with partial mitochondrial membrane depolarization under sustained GSH depletion. Quantitative flow cytometry analysis of TMRE fluorescence confirmed and extended these microscopy observations (Fig. 3H–K). In iron-treated cultures, all iron-exposed groups (AI50, AI100, CI50, CI100) showed elevated TMRE median fluorescence intensity (MFI) relative to untreated controls, and this elevation reached statistical significance across the iron-treated group (*p < 0.05) (Fig. 3H, I). This elevation in TMRE signal under both acute and chronic iron loading, occurring in parallel with significantly elevated mitochondrial superoxide production under chronic conditions (Fig. 3D), indicates that iron exposure drives a state of increased mitochondrial membrane potential rather than membrane depolarization, with the effect most pronounced under chronic exposure. In BSO-treated cultures, neither acute dose of BSO (AB25, AB50) significantly altered TMRE MFI relative to untreated controls (Fig. 3J, K). Chronic 25 μM BSO (CB25) produced a statistically significant reduction in TMRE fluorescence (*p < 0.05), while chronic 50 μM BSO (CB50) showed a consistent downward trend in the same direction that did not achieve statistical significance. Thus, the direction of TMRE changes under BSO treatment was opposite to that observed under iron treatment, despite both conditions producing elevated mitochondrial superoxide.

### Chronic iron exposure and GSH depletion enhance lipid peroxidation compared to acute insults

**Fig. 4:**
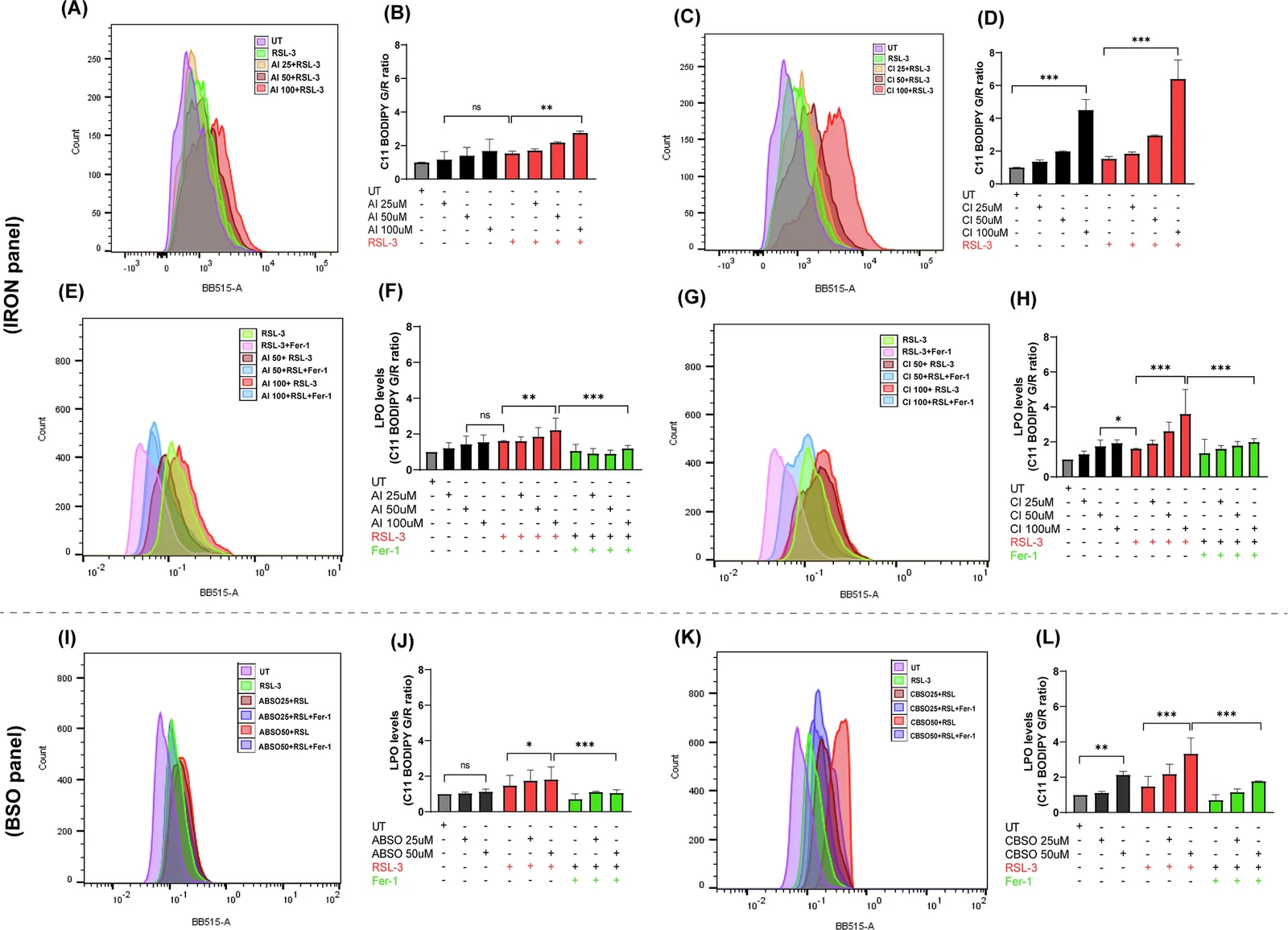
Chronic iron exposure and GSH depletion enhance lipid peroxidation as compared to acute insults. **A** Representative BODIPY-C11 flow-cytometry histograms illustrating lipid peroxidation (LPO) following acute iron exposure (25–100 µM) with or without RSL-3. **B** Quantification of the BODIPY 581/591 green:red fluorescence ratio showing that acute iron alone does not significantly alter LPO whereas co-treatment with RSL-3 (100 nM, 3 h) significantly increases lipid peroxidation (**p < 0.01). **C** Representative histograms following chronic (9-day) iron exposure with or without RSL-3. **D** Quantification showing that chronic iron exposure substantially elevates LPO as compared with acute treatment with the largest effect at 100 µM and that RSL-3 (100 nM, 3 h) further amplifies this increase (***p < 0.001), indicating that sustained iron loading primes cells for ferroptosis-associated lipid damage. **E**, **G** Representative histograms showing the effect of ferrostatin-1 (Fer-1) co-treatment under acute and chronic iron conditions. **F**, **H** Quantification demonstrating that Fer-1 significantly attenuates RSL-3- and iron-induced LPO under both acute and chronic paradigms (***p < 0.001). **I** Representative histograms showing LPO following acute BSO (25 or 50 µM) with RSL-3 and Fer-1. **J** Quantification showing that acute BSO alone does not significantly change LPO whereas co-treatment with RSL-3 (100 nM, 3 h) causes a mild but significant increase (*p < 0.05) that is fully reversed by Fer-1 (***p < 0.001). **K** Representative histograms following chronic BSO (25 or 50 µM) with RSL-3 and Fer-1. **L** Quantification demonstrating that chronic BSO significantly increases LPO (**p < 0.01), which is further enhanced by RSL-3 (100 nM, 3 h) (***p < 0.001) and that Fer-1 co-treatment markedly reduces LPO under both chronic BSO alone and BSO + RSL-3 conditions (***p < 0.001). Bars represent mean ± SEM (*n* = 3). Color codes for all flow cytometry histograms in this figure are as follows: UT, magenta; RSL-3 alone, green; lower iron or BSO dose combined with RSL-3 (AI25, CI25, ABSO25, or CBSO25), orange for iron panels and dark red for BSO panels; middle iron dose combined with RSL-3 (AI50 or CI50), dark red; higher iron or BSO dose combined with RSL-3 (AI100, CI100, ABSO50, or CBSO50), red; all Fer-1-containing conditions, sky blue. The same color assignment is maintained across all panels within this figure. UT untreated, AI acute iron, CI chronic iron, ABSO acute BSO, CBSO chronic BSO, RSL-3 RAS-selective lethal 3, Fer-1 ferrostatin-1, LPO lipid peroxidation, G/R green-to-red fluorescence ratio, ns not significant.

Given the suppression of GPX4 and xCT under chronic iron, we next evaluated whether the temporal dimension of iron treatment affects lipid peroxidation (LPO), a hallmark of ferroptosis and, potentially, ferroptotic stress. As shown in Fig. 4A, B, acute iron exposure alone (25–100 µM) for 8 h did not significantly alter the BODIPY 581/591 fluorescence ratio whereas co-treatment with the ferroptosis inducer RSL-3 (100 nM for 3 h) significantly increased lipid peroxidation (**p < 0.01). However, under chronic iron exposure (Fig. 4C, D), LPO levels were substantially elevated even in the absence of RSL-3, with the effect most pronounced at 100 µM iron. These levels were further amplified by RSL-3 treatment (***p < 0.001) indicating that chronic iron loading primes cells to drive the accumulation of ferroptosis-associated lipid peroxides. As expected, co-treatment with the ferroptosis inhibitor ferrostatin-1 (Fer-1) significantly attenuated RSL-3- and iron-induced LPO under both acute and chronic paradigms (Fig. 4E–H, ***p < 0.001). These findings demonstrate that prolonged iron exposure creates a lipid peroxidation-prone state that is sensitive to ferroptosis inducers.

Because chronic iron exposure reduces GSH levels (as shown in Fig. 3A), we next asked whether direct GSH depletion using BSO produces a similar lipid peroxidation phenotype, independent of exogenous iron supplementation. As shown in Fig. 4I–L acute BSO alone did not significantly alter LPO, while co-treatment with RSL-3 caused a mild increase (*p < 0.05) that was reversed by Fer-1 (***p < 0.001). Chronic BSO exposure markedly increased LPO (**p < 0.01) on its own which was further enhanced by RSL-3 (***p < 0.001) and both effects were mitigated by Fer-1 (***p < 0.001). Together, these findings indicate that sustained but not transient disruption of either iron or GSH homeostasis increases cellular susceptibility to lipid peroxidation and potentially ferroptosis, as reflected by the stronger lipid peroxidation response to RSL-3 during chronic as compared to acute treatment.

### Chronic iron and GSH depletion sensitize cells to lipid peroxidation induced by non-ferroptotic oxidative stress

**Fig. 5:**
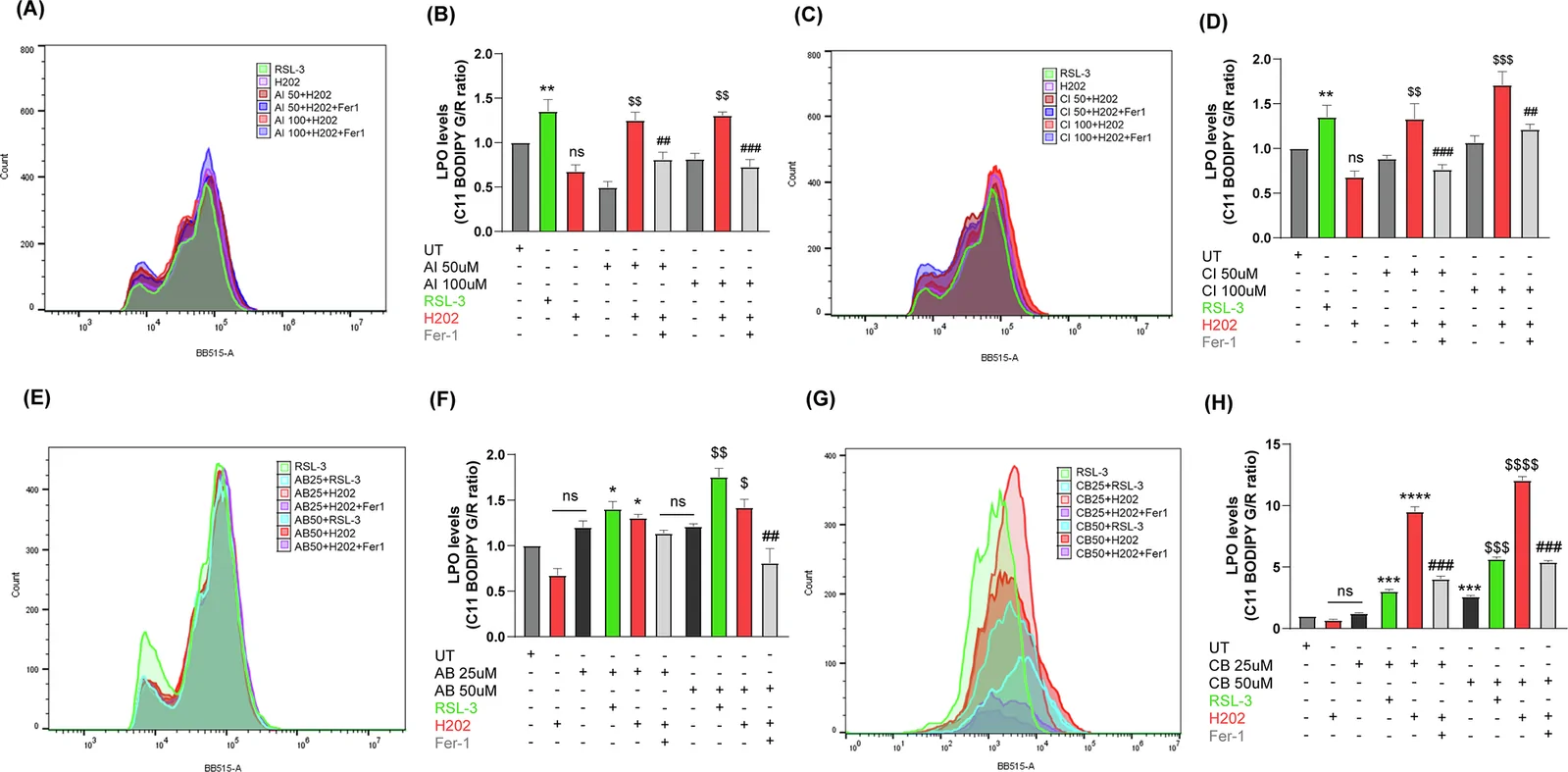
Chronic iron accumulation and GSH depletion sensitize cells to additional oxidative stresses leading to enhanced lipid peroxidation. **A** Representative BODIPY-C11 histograms showing LPO in acutely iron-treated cells exposed to RSL-3, H₂O₂, and Fer-1 in the indicated combinations. **B** Quantification of LPO normalized to untreated controls showing that RSL-3 (300 nM, 3 h) alone significantly increases LPO and that the combination of acute iron and H₂O₂ (300 µM, 1 h) produces a notable rise in LPO ($$*p* < 0.01), which is reduced by Fer-1 (##*p* < 0.01). **C** Representative BODIPY-C11 histograms for chronically iron-treated cells. **D** Quantification showing that chronic iron exposure combined with H₂O₂ (300 µM, 1 h) causes a markedly greater increase in LPO, particularly at 100 µM ($$$*p* < 0.001), and that Fer-1 co-treatment significantly reduces this elevation (##*p* < 0.01). **E** Representative histograms showing LPO in acutely BSO-treated cells (25 and 50 µM) with RSL-3, H₂O₂, and Fer-1. **F** Quantification indicating that acute BSO produces only a mild LPO increase which becomes significant when combined with RSL3 or H₂O₂ (300 µM, 1 h) (*p < 0.05) and is attenuated by Fer-1 (##*p* < 0.01). **G** Representative histograms showing LPO in chronically BSO-exposed cells under the same treatment combinations. **H** Quantification showing a robust increase in LPO when chronic BSO is combined with RSL-3 (300 nM, 3 h) or H₂O₂ (300 µM, 1 h) (****p < 0.0001, $$$$*p* < 0.0001), both of which are substantially reduced by Fer-1 co-treatment (###*p* < 0.001), indicating that chronic ferroptotic stress enables general oxidants to drive iron-dependent lipid peroxidation. Bars represent mean ± SEM (*n* = 3). Color codes for all flow cytometry histograms in this figure are as follows. Iron panels (**A** and **C**): RSL-3 alone, green; H_2_O_2_ alone, magenta; lower iron dose combined with H_2_O_2_ (AI50 or CI50), dark red; lower iron dose combined with H_2_O_2_ and Fer-1 (AI50 + H_2_O_2_ + Fer-1 or CI50 + H_2_O_2_ + Fer-1), blue; higher iron dose combined with H_2_O_2_ (AI100 or CI100), red; higher iron dose combined with H_2_O_2_ and Fer-1 (AI100 + H_2_O_2_ + Fer-1 or CI100 + H_2_O_2_ + Fer-1), sky blue. BSO panels (**E** and **G**): RSL-3 alone, green; lower BSO dose combined with RSL-3 (AB25 + RSL-3 or CB25 + RSL-3), cyan; lower BSO dose combined with H_2_O2 (AB25 + H2O2 or CB25 + H2O2), red; lower BSO dose combined with H_2_O_2_ and Fer-1 (AB25 + H_2_O_2_ + Fer-1 or CB25 + H_2_O_2_ + Fer-1), magenta; higher BSO dose combined with RSL-3 (AB50 + RSL-3 or CB50 + RSL-3), dark cyan; higher BSO dose combined with H_2_O_2_ (AB50 + H_2_O_2_ or CB50 + H_2_O_2_), red; higher BSO dose combined with H_2_O_2_ and Fer-1 (AB50 + H_2_O_2_ + Fer-1 or CB50 + H_2_O_2_ + Fer-1), magenta. The same color assignment is maintained between acute and chronic panels within each treatment group. Note that the y-axis scale in panel (**H**) extends to 15, reflecting the substantially greater magnitude of lipid peroxidation observed under chronic BSO conditions relative to all other treatment groups in this figure. UT untreated, AI acute iron, CI chronic iron, AB acute BSO, CB chronic BSO, RSL-3 RAS-selective lethal 3, Fer-1 ferrostatin-1, H_2_O_2_ hydrogen peroxide, LPO lipid peroxidation, G/R green-to-red fluorescence ratio, ns not significant.

We next asked if chronic ferroptotic stress also increases lipid peroxidation induced by general oxidative insults such as hydrogen peroxide as compared to acute treatments. Cells under acute or chronic stress (8 h or 9 days) were challenged with RSL-3 as a positive control (300 nM for the last 3 h) or hydrogen peroxide (300 μM for the last hour). These concentrations allowed for the detection of sensitization induced by chronic iron or BSO pretreatment while inducing minimal toxicity when administered alone. Under acute iron conditions (Fig. 5A, B), RSL-3 treatment significantly increased LPO, as expected. When acutely iron-treated cells were challenged with H₂O₂, we observed an increase in LPO ($$*p* < 0.01). This effect was suppressed by Fer-1 (##*p* < 0.01). In chronically iron-treated cells (Fig. 5C, D), H₂O₂ co-treatment caused a markedly greater increase in LPO, particularly at 100 µM ($$$*p* < 0.001), which was again suppressed by Fer-1 (*##p* < 0.01). This demonstrates that, as compared with acute exposure, chronic iron at higher concentrations allows for a significantly greater sensitization of cells to general oxidative stress.

We next tested whether the temporal dimension of BSO treatment produces a similar differential sensitization to H_2_O_2_-induced LPO. As shown in Fig. 5E–H, acute BSO treatment produced only a mild LPO rise with H₂O₂ (*p < 0.05) whereas chronic BSO exposure caused a pronounced increase when combined with either RSL-3 or H₂O₂ (note different y-axis scales ****p < 0.0001, $$$*p* < 0.0001) which were both prevented by Fer-1 (###*p* < 0.001). The difference between chronic and acute BSO treatment on H_2_O_2_-induced LPO paralleled the temporal pattern observed with iron. The ability of Fer-1 to block H₂O₂-induced lipid peroxidation under chronic treatments shows how sustained ferroptotic stress allows for non-ferroptotic oxidants to drive iron-dependent lipid peroxidation. Thus, cells chronically stressed by iron accumulation or GSH depletion not only exhibit increased susceptibility to ferroptosis-associated induction of LPO but also increased susceptibility to LPO induced by additional oxidative challenges that by themselves do not induce ferroptosis [41] in a way that is profoundly different from the acute stresses that are generally used in in vitro models, thereby more closely reproducing the progressive redox vulnerability seen in neurodegenerative diseases.

### Chronic iron accumulation and GSH depletion promote ferroptotic cell death

**Fig. 6:**
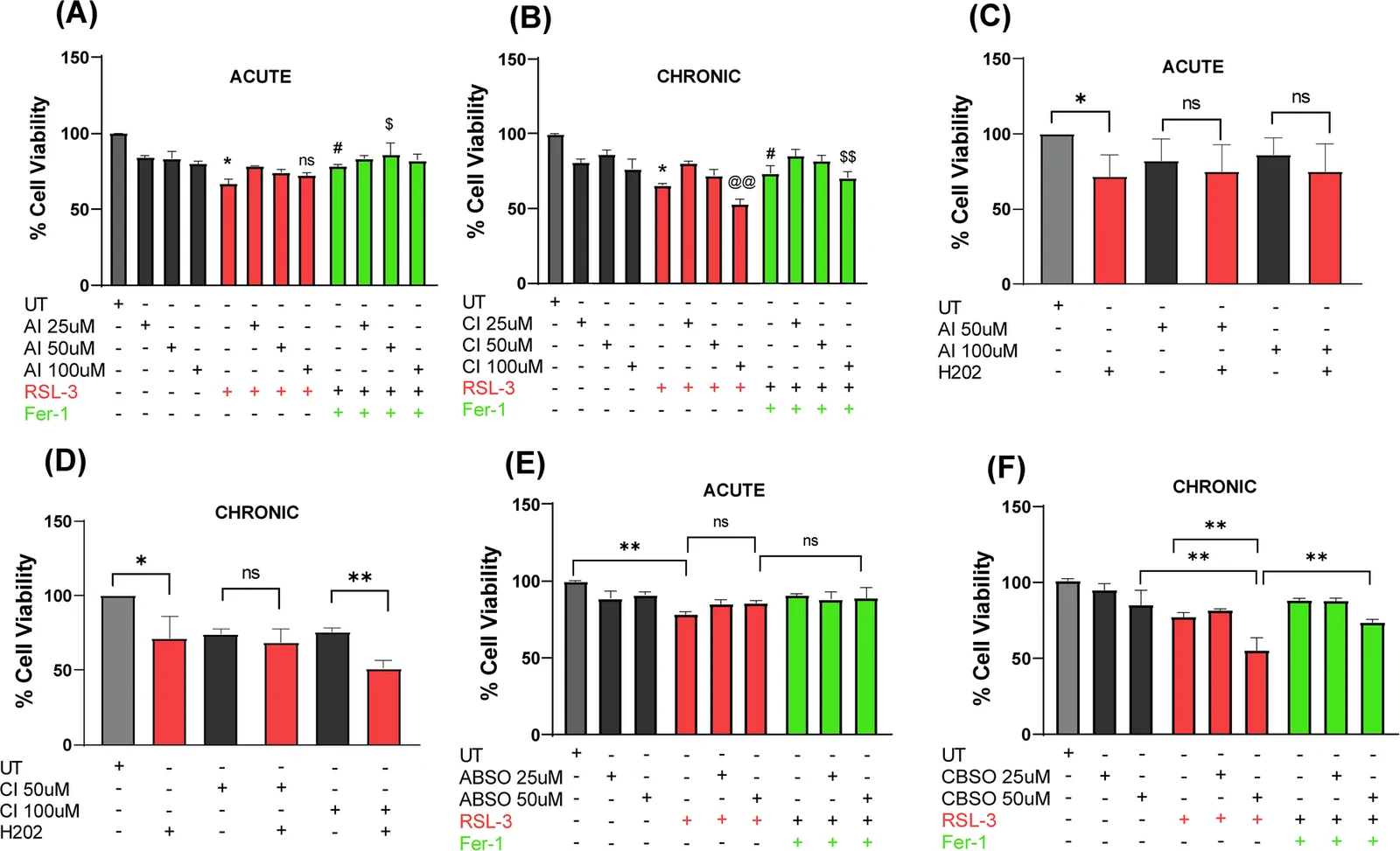
Chronic iron accumulation and GSH depletion increase vulnerability to oxidative damage and ferroptotic cell death. **A**–**F** Cell viability was assessed by the MTT assay following acute and chronic exposure to iron or BSO with RSL-3, H₂O₂, and Fer-1 as indicated. **A** Acute iron exposure (with or without RSL-3) does not significantly reduce cell viability; RSL-3 alone (50 nM, 16 h) produces a modest but significant decrease in viability (*p < 0.05). **B** Chronic 100 µM iron combined with RSL-3 (50 nM, 16 h) markedly decreases viability (@@*p* < 0.01) and this decrease is rescued by Fer-1 ($$*p* < 0.01). **C** Under acute iron conditions, H₂O₂ (300 µM, 1 h) alone causes a modest but significant reduction in viability (*p < 0.05) and co-treatment with acute iron does not further reduce survival. **D** In contrast, chronic 100 µM iron combined with H₂O₂ (300 µM, 1 h) significantly decreases viability (**p < 0.01) compared with either treatment alone, indicating sensitization to H₂O₂-induced cell death under chronic iron loading. **E** Acute BSO exposure, alone or with RSL-3 (50 nM, 16 h), does not significantly affect cell viability. **F** Chronic BSO combined with RSL-3 (50 nM, 16 h) markedly reduces survival (**p < 0.01), and Fer-1 co-treatment maintains viability (**p < 0.01). RSL-3 alone induces moderate cell death that is also reversed by Fer-1, confirming ferroptotic cell death. Bars represent mean ± SEM (*n* = 3).

To determine whether the temporal dimension of ferroptotic stress translates into differential effects on cell viability, we compared cell survival under acute versus chronic exposure paradigms using the MTT assay. Acute iron exposure had no significant effect on viability (Fig. 6A). RSL-3 (50 nM for 16 h) produced a modest but significant decrease in viability (*p < 0.05) which was not increased by acute iron exposure. In contrast, while chronic exposure to 100 µM iron alone also did not significantly affect viability, chronic iron combined with RSL-3 markedly reduced survival (*@@p* < 0.01) and this loss was rescued by Fer-1 (*$$p* < 0.01) (Fig. 6B). The resistance to cell death under acute conditions versus the marked sensitivity under chronic conditions suggests that the duration of iron exposure, not merely its concentration, contributes to vulnerability to ferroptotic death.

H₂O₂ alone caused a modest but significant reduction in viability (*p < 0.05) (Fig. 6C) and acute iron combined with H₂O₂ did not significantly differ from H₂O₂ alone. In contrast, treatment of chronic 100 µM iron cells with H₂O₂ significantly decreased viability (**p < 0.01) compared to either treatment alone (Fig. 6D) demonstrating that chronic but not acute iron exposure also sensitizes cells to H₂O₂-induced cell death.

To determine if the temporal dimension of GSH depletion is sufficient to establish differential vulnerability independent of exogenous iron, we examined cell viability under acute versus chronic BSO stress (Fig. 6E, F). Acute treatment with BSO, alone or with RSL-3, did not alter viability, but chronic BSO exposure combined with RSL-3 caused a strong decrease in viability (**p < 0.01), that was prevented by Fer-1 (**p < 0.01). Together, these data show that prolonged iron accumulation or GSH depletion establishes a vulnerable redox state that converts otherwise tolerable oxidative challenges into ferroptotic cell death, emphasizing once again the pathogenic potential of chronic ferroptotic stress in neuronal cells as compared to short term insults.

## Discussion

The central finding of this study is that sustained disruption of iron or GSH homeostasis induces a stable, non-lethal cellular state that we term chronoferroptosis (chronic ferroptotic stress): a persistent ferroptotic adaptation characterized by coordinated molecular alterations including ferritin upregulation, suppression of GPX4, xCT, and STEAP-3, elevated mitochondrial ROS, and increased lipid peroxidation, that is fundamentally different from the more frequently used short-term treatments. Importantly, cells maintain viability under prolonged stress alone yet exhibit significantly increased vulnerability to additional oxidative insults, including both the ferroptosis inducer RSL-3 and the general oxidant H₂O₂. We believe that this represents a mechanistically distinct intermediate condition between normal cellular homeostasis and ferroptotic cell death-a state of ‘primed vulnerability’ in which ferroptosis defense mechanisms are chronically compromised but remain functionally sufficient to prevent death. Chronoferroptosis could explain the prolonged prodromal period between initial iron accumulation and GSH depletion and eventual neuronal loss in neurodegenerative diseases, thereby defining a critical therapeutic window for early intervention before cells transition to irreversible degeneration. The temporal dimension of iron and GSH disruption appears to determine cellular vulnerability to ferroptosis. Chronic exposure (9 days) produced coordinated molecular changes that were minimal or absent under acute exposure (6–8 h) despite identical concentrations. This temporal dependence reveals a critical gap in the ferroptosis literature. The predominant use of acute exposure models (typically 4–24 h with erastin, RSL-3, or conditional GPX4 deletion) captures the terminal execution phase of ferroptosis but fails to model the progressive metabolic remodeling that occurs during sustained stress. Our chronic paradigm demonstrates that neuronal cells can survive under prolonged oxidative pressure while undergoing a gradual transformation from a stress-resistant to a stress-vulnerable state, a transition bypassed entirely in acute models that trigger immediate cell death. This distinction is relevant for understanding neurodegenerative diseases where iron accumulation and GSH depletion occur over years or decades, not hours or days [34].

Chronic iron exposure produced pronounced ferritin heavy chain (FTH1) induction accompanied by reduced labile iron, consistent with adaptive sequestration of redox-active iron [42]. In contrast, acute iron treatment transiently increased labile iron prior to ferritin upregulation. This temporal distinction demonstrates that short-term iron loading fails to capture the iron-storage response missed in acute studies terminating within 24 h. The sustained decrease in labile iron under chronic conditions likely results from combined ferritin sequestration and downregulation of STEAP-3, an endosomal ferrireductase that releases Fe²⁺ into the cytosol [22], limiting redox-active iron available for Fenton chemistry while potentially restricting cellular iron bioavailability [43]. It is important to note that while our model demonstrates ferritin-mediated iron sequestration, this represents only one aspect of age-related iron dysregulation. In aging brain, total iron continues to accumulate despite ferritin upregulation [5] suggesting that chronic iron exposure in vivo may eventually overwhelm storage capacity. Our findings model an early adaptive phase that precedes iron homeostasis breakdown in advanced neurodegeneration.

Importantly, chronic BSO exposure also induced ferritin upregulation in the absence of external iron supplementation. Since GSH can function as an iron chelator, its depletion by chronic BSO may liberate GSH-bound iron, triggering compensatory ferritin expression [44, 45]. Despite this adaptation, chronic BSO resulted in persistent increases in mitochondrial ROS and lipid peroxidation indicating that ferritin upregulation alone cannot prevent oxidative damage when GSH is depleted. Interestingly, chronic iron exposure also reduced GSH levels while acute exposure did not, suggesting that sustained increases in iron compromise GSH homeostasis through oxidative consumption. This establishes a critical mechanistic link whereby chronic iron reduces GSH levels and chronic GSH depletion triggers iron redistribution with both converging on ferroptotic vulnerability.

Among the molecular changes found in response to alterations in iron or GSH, the decrease in the levels of GPX4 may represent a crucial shift in the antioxidant defense landscape [25, 46]. Acute exposure to either insult produced mild GPX4 reduction, whereas chronic exposure caused pronounced decline. Thus, although these stressors act through distinct biochemical routes, they converge on the same protective node.

Lipid peroxidation serves as both a marker and a contributor to the oxidative vulnerability that characterizes chronic ferroptotic stress. While acute exposures to iron or BSO produced only minor increases in lipid peroxidation, indicating that intact antioxidant systems can buffer transient stress, prolonged iron loading or GSH depletion caused sustained lipid peroxidation, which intensified upon challenge with RSL-3 or hydrogen peroxide. The amplified response in chronically treated cells suggests the development of a primed state for lipid oxidative damage. Ferrostatin-1 effectively suppressed lipid peroxidation induced by chronic stress alone and when combined with RSL-3 or H_2_O_2_. Critically, Fer-1 also restored cell viability under conditions where chronic iron or BSO pretreatment combined with RSL-3 or H_2_O_2_ caused significant cell death, confirming that lipid peroxidation is a principal mechanism linking chronic redox imbalance to ferroptotic vulnerability. The accumulation of lipid peroxides under chronic conditions illustrates how persistent iron overload or GSH depletion, despite initial compensatory ferritin upregulation, ultimately creates a pro-degenerative trajectory [47, 48].

Mitochondria are both sources and amplifiers of reactive oxygen species during ferroptosis, and chronic stress can convert them from protective to pro-oxidant organelles [49]. Both chronic iron exposure and chronic BSO treatment elevated mitochondrial ROS production further contributing to the oxidative imbalance that characterizes this vulnerable state. Assessment of mitochondrial membrane potential by TMRE revealed that iron exposure and BSO-induced GSH depletion produced distinct effects on ΔΨm. Iron exposure significantly increased TMRE fluorescence relative to untreated controls, regardless of the treatment duration. Increased mitochondrial membrane potential has been reported during ferroptotic conditions involving increased mitochondrial oxidative phosphorylation activity [50], and the elevation of the TMRE signal observed here under iron loading is consistent with this previously described pattern. In contrast, chronic BSO treatment showed a very different pattern. CB25 produced a statistically significant reduction in TMRE fluorescence, while CB50 showed a downward trend that did not reach statistical significance. GSH is an important component of mitochondrial antioxidant defense, and its sustained depletion has been associated with mitochondrial dysfunction [40]. The observation that chronic GSH depletion by BSO reduced rather than elevated mitochondrial membrane potential, while both treatments elevated mitochondrial superoxide, suggests that iron loading and direct GSH depletion engage mitochondria through distinct mechanisms under chronic ferroptotic stress conditions. This apparent paradox is consistent with published evidence demonstrating that sustained GSH depletion in brain mitochondria impairs Complex I function and elevates mitochondrial ROS production concurrent with reduced membrane potential [51, 52], a pattern in which electron transport chain dysfunction drives superoxide generation through mechanisms that are dissociated from the electrochemical gradient, rather than arising as a consequence of hyperpolarization as observed under iron loading.

Nevertheless, despite all of these oxidative alterations, cells remained viable under chronic iron or BSO exposure alone. However, when chronically stressed cells were challenged with RSL-3 or hydrogen peroxide, cell viability declined significantly. These findings demonstrate that persistent disruption of iron or GSH homeostasis establishes a stable yet highly vulnerable state to additional oxidative insults. Our results show that sustained iron accumulation and/or GSH depletion progressively remodel neuronal redox balance into chronic ferroptotic stress which is fundamentally different from acute treatments. In neurodegenerative diseases, such a sustained imbalance likely emerges well before cell death, defining an early window for therapeutic intervention.

### Limitations of the study

While this study employed RA-differentiated SH-SY5Y cells that recapitulate key neuronal redox processes and enable temporal resolution of adaptive responses, in vivo validation is necessary to establish whether these chronic adaptations also occur within the intact brain microenvironment where glial-neuronal interactions and systemic iron regulation may modulate ferroptotic vulnerability.

## Conclusion and future directions

This study demonstrates that sustained, but not transient, disruption of iron and GSH homeostasis induces coordinated cellular changes consistent with ferroptotic stress in differentiated neuronal cells. The duration of exposure, rather than concentration alone, determined cellular outcomes as acute treatments (6–8 h) produced minimal alterations whereas chronic exposure (9 days) to identical iron or BSO concentrations elicited multiple changes associated with ferroptosis. Thus, these findings reveal a temporal dimension to ferroptotic vulnerability that is not captured by conventional acute models which typically induce rapid cell death within hours. The chronic stress paradigm employed here more closely approximates the prolonged timescale of iron accumulation and GSH depletion observed in the aging brain and in neurodegenerative diseases. Importantly, direct GSH depletion via BSO recapitulated the iron-induced phenotype demonstrating that GSH loss, whether arising from iron-mediated oxidative consumption or biosynthetic blockade, is a critical mediator of this vulnerable state. Future investigations will be focused on identifying additional molecular markers that distinguish chronoferroptosis and testing whether interventions targeting iron homeostasis or GSH restoration can reverse or prevent progression to cell death, potentially establishing chronoferroptosis as a therapeutic target in age-related neurodegeneration.

## Materials and methods

### Reagents

The materials and reagents employed in this study were procured from the following suppliers: Dulbecco’s Modified Eagle’s Medium F12 (DMEM:F12), Penicillin-Streptomycin Solution, and Fetal Bovine Serum (FBS) were obtained from Invitrogen (Invitrogen, Carlsbad, CA, USA). Assay kits and solutions, including the Pierce BCA Protein Assay Kit, Cell Lysis Buffer, and SuperSignal West Pico PLUS Chemiluminescent Substrate, were sourced from Thermo Fisher Scientific (Thermo Fisher Scientific Inc., Waltham, MA, USA). The chemical reagents retinoic acid (RA), MTT, ferrous sulfate (FeSO₄), buthionine sulfoximine (BSO) and carbonyl cyanide 3-chlorophenylhydrazone (CCCP; cat. no. C2759) were procured from Sigma (Sigma-Aldrich Corporation, St. Louis, MO, USA). Tetramethylrhodamine ethyl ester (TMRE; cat. no. T669) and Hoechst 33342 (cat. no. H1399) were from Thermo Fisher Scientific (Waltham, MA, USA). The Nunc™ Lab-Tek™ 8-well Chamber Slide System was from Thermo Fisher Scientific (cat. no. 155409).

### Cell culture and differentiation

SH-SY5Y cells were a kind gift from Prof. R. Luke Wiseman (Scripps Research, La Jolla, CA). Cells were cultured in DMEM:F12 medium supplemented with 10% FBS and 1% penicillin-streptomycin and maintained at 37 °C in a humidified 10% CO₂ incubator. The medium was changed on alternate days. Cells were differentiated with retinoic acid (RA) as described previously [53] to induce neuronal-like characteristics. Briefly, cells were seeded in plates/dishes and treated with 10 µM RA for 8 days. During differentiation, the FBS concentration was reduced from 10% to 2% to promote neuronal maturation, growth arrest, and morphological differentiation.

### Dose-response optimization and exposure paradigms

An extensive dose-response analysis was performed to establish iron and GSH depletion ranges that induce oxidative alterations without significant cell loss. RA-differentiated SH-SY5Y cells were treated with FeSO₄ (1–500 µM) and BSO (5–300 µM) for up to 9 days. Concentrations producing clear biochemical changes while maintaining >85% cell viability were selected for subsequent experiments. From this optimization, 50 µM and 100 µM FeSO₄ and 25 µM and 50 µM BSO were chosen as working doses. In certain initial experiments, 25 µM FeSO₄ was also used to confirm dose responsiveness at the lower threshold. For chronic exposure, RA-differentiated cells were treated continuously for 9 days, with alternate day replacement of medium containing fresh RA and the appropriate FeSO₄ or BSO concentration to ensure constant exposure. For acute treatment, cells underwent RA differentiation under identical conditions but received the same concentrations of FeSO_4_ or BSO for only 8 h on day 9. This design allowed direct comparison of short-term (acute) and sustained (chronic) oxidative stress responses under identical culture conditions.

### Cell viability assay

Cells were seeded at 2 × 10³ cells per well in 96-well plates and differentiated for 8 days with RA, while being subjected to FeSO₄ or BSO under chronic (9-day continuous exposure with media replacement every 2 days) or acute (8-h exposure on day 9) conditions. Following the chronic or acute iron/BSO treatments, cells were challenged on day 9 with RSL-3 or H₂O₂ before viability assessment. These RSL-3 and H₂O₂ concentrations were selected to be minimally toxic when administered alone, allowing detection of sensitization induced by chronic pretreatment. After treatment, MTT (2.5 mg/mL) was added and incubated for 4 h at 37 °C. The supernatant was removed, formazan crystals were solubilized, and absorbance was measured at 570 nm on a SpectraMax M5 microplate reader. Cell viability was expressed as a percentage of untreated control values. Prior to adding the MTT reagent, the plates were examined microscopically to assess viability visually.

### Total GSH (tGSH) measurement

To quantify total GSH (tGSH), 3 × 10⁴ SH-SY5Y cells were seeded in 60 mm dishes. The chronic treatment group was exposed to varying concentrations of iron for 9 days, whereas the acute and untreated control groups underwent identical handling and media changes but without iron exposure. On day 9, the acute group received iron or BSO treatment for 8 h. Following treatment, cells were washed with ice-cold PBS, scraped, and immediately mixed with sulfosalicylic acid (SSA) to achieve a final concentration of 3.3%, as described previously [54], to deproteinize samples and stabilize GSH. tGSH levels were quantified using an enzymatic recycling assay based on the reduction of 5,5′-dithiobis(2-nitrobenzoic acid) (DTNB) by GSH reductase in the presence of NADPH, producing a chromophore measured spectrophotometrically at 412 nm. Values were normalized to total protein content obtained from the acid-precipitated pellet, which was solubilized in 0.2 N NaOH at 37 °C overnight and quantified using the bicinchoninic acid (BCA) protein assay (Pierce, Rockford, IL, USA).

### Labile iron pool measurement by flow cytometry

The intracellular labile iron pool (LIP) was quantified in RA-differentiated SH-SY5Y cells using FerroRed dye (Dojindo Molecular Technologies, Kumamoto, Japan) via flow cytometry. Cells were seeded in 6-well plates and subjected to chronic (9-day) or acute (8-h) treatments with FeSO₄ (50 or 100 µM) or BSO (25 or 50 µM). Following treatment, cells were washed with Hank’s Balanced Salt Solution (HBSS) and incubated with 1 µM FerroRed for 30 min at 37 °C in the dark. Stained cells were washed twice with HBSS and analyzed on a BD A3 flow cytometer (BD Biosciences, Franklin Lakes, NJ, USA) using standard settings (Ex/Em= 488/585 nm). A minimum of 10,000 single-cell events were recorded per sample. Median fluorescence intensity (MFI) values were calculated after background subtraction from unstained controls using FlowJo software.

### Lipid peroxidation measurement by flow cytometry

Lipid peroxidation was assessed in RA-differentiated SH-SY5Y cells after chronic (9 days) or acute (8 h) exposure to iron or BSO, alone or in combination with a short-term treatment of RSL-3 or H₂O₂. Where indicated, ferrostatin-1 (Fer-1; 1 µM) was added concurrently with RSL-3 or H₂O₂. After treatment, cells were incubated with 2 µM BODIPY-C11 (Thermo Fisher Scientific, Waltham, MA, USA) for 30 min at 37 °C in the dark, washed with PBS, trypsinized, and resuspended in ice-cold HBSS supplemented with 10% FBS. Flow cytometry was performed on a BD A3 flow cytometer with excitation/emission settings optimized for BODIPY-C11 (581/591 nm for reduced form, 488/510 nm after oxidation). Lipid peroxidation was quantified as the red-to-green fluorescence ratio using FlowJo software.

### Mitochondrial membrane potential measurement by flow cytometry and airyscan microscopy

Mitochondrial membrane potential (ΔΨm) was assessed in RA-differentiated SH-SY5Y cells subjected to chronic (9-day continuous exposure with media replacement every 2 days) or acute (8 h) treatments with FeSO₄ (50 or 100 µM) or BSO (25 or 50 µM), alongside untreated (UT) controls and a positive control for mitochondrial depolarization employing 20 µM CCCP (carbonyl cyanide 3-chlorophenylhydrazone; Sigma-Aldrich, St. Louis, MO, USA), using tetramethylrhodamine ethyl ester (TMRE; Thermo Fisher Scientific, Waltham, MA, USA). Cells were seeded in 24-well plates for flow cytometry or Nunc™ Lab-Tek™ 8-well Chamber Slide System glass-bottom chambers for microscopy and were differentiated and treated as described under Cell Culture and Differentiation. For flow cytometry, treated cells from 24-well plates were washed twice with Hank’s Balanced Salt Solution (HBSS), incubated with 50 nM TMRE for 30 min at 37 °C in the dark, washed twice with HBSS, trypsinized, and resuspended in HBSS supplemented with 10% FBS. Samples were analyzed immediately on a BD A3 flow cytometer (BD Biosciences, Franklin Lakes, NJ, USA) using standard settings (Ex/Em = 488/575 nm), recording a minimum of 10,000 single-cell events per sample. Median fluorescence intensity (MFI) values were calculated after background subtraction from unstained controls using FlowJo software. For Airyscan microscopy, cells in Nunc™ Lab-Tek™ 8-well chamber slides were washed once with pre-warmed phenol red-free DMEM (37 °C), then directly incubated with 50 nM TMRE in phenol red-free DMEM for 30 min at 37 °C in the dark (no post-staining wash to preserve ΔΨm and enable immediate imaging). Hoechst 33342 (1 µg/mL; Thermo Fisher Scientific) was added during the final 10 min of TMRE incubation for nuclear counterstaining. Chambers were then directly transferred to the Zeiss LSM880 Airyscan microscope stage (maintained at 37°C in a humidified 5–10% CO₂ chamber) and imaged live using an alpha Plan-Apochromat 63×/1.4 oil immersion objective (Ex/Em = 561/570–620 nm). Laser power was minimized to prevent photobleaching with pixel dwell times of 4–9 µs and zoom factors adjusted for optimal resolution at each magnification. Raw Airyscan images were processed using ZEN software for super-resolution reconstruction.

### Cell lysate preparation and immunoblotting

Western blotting was performed following a modified protocol based on ref. [55]. SH-SY5Y cells were lysed in ice-cold RIPA buffer (20 mM Tris-HCl, pH 7.4, 150 mM NaCl, 5 mM EDTA, 1% IGEPAL-CA-630, 3 mM Na_3_VO_4_, 3 mM PMSF), supplemented with 1% protease inhibitor cocktail and 1% phosphatase inhibitor cocktail. The cell lysates were incubated for 20–25 min on ice followed by centrifugation at 14,000 rpm for 15 min at 4 °C. The resulting supernatant was collected and stored at −20 °C for future use. Protein concentrations were determined using the Pierce BCA Protein Assay Kit (Thermo Fisher Scientific Waltham, MA) according to the manufacturer’s instructions. For Western blot analysis, 30–40 μg of protein was loaded onto 4–12% gradient Criterion XT Precast Bis-Tris Gels (Bio-Rad, Hercules, CA) and transferred onto PVDF membranes using a Trans-Blot Turbo System (Bio-Rad, Hercules, CA). Membranes were blocked with 5% skim milk in TBST (20 mM Tris buffer, pH 7.5, 0.5 M NaCl, 0.1% Tween 20) for 1 h at room temperature. After blocking, membranes were incubated overnight at 4 °C with primary antibodies diluted in TBST. Primary antibodies used were: anti-GPX4 (Santa Cruz Biotechnology, Inc., Dallas, TX, USA; catalog #sc-166570; 1:500 dilution), anti-FTH1 (Cell Signaling Technology, Inc., Danvers, MA, USA; catalog #3998; 1:1000 dilution), anti-xCT/SLC7A11 (catalog #26864-1-AP; 1:2000 dilution) and anti-STEAP-3 (catalog #28478-1-AP; 1:1000 dilution) from Proteintech Group, Inc., Rosemont, IL, USA. GPX4 was incubated in 5% non-fat milk in TBST, while all other antibodies were incubated in 5% BSA in TBST. Following washing with TBST, membranes were incubated with horseradish peroxidase-conjugated goat anti-rabbit or anti-mouse secondary antibodies (Bio-Rad, Hercules, CA) diluted 1:5000 in 5% skim milk in TBST. After secondary antibody incubation, membranes were washed three times for 15 min each. Protein bands were visualized using the SuperSignal West Pico PLUS Chemiluminescent Substrate and imaged with a ChemiDoc MP Imaging System (Bio-Rad, Hercules, CA). Band intensities were analyzed by densitometry using ImageJ software (Version 1.54f, NIH, USA). For each sample, the optical density of the target protein band was normalized to the optical density of the corresponding GAPDH loading control band from the same biological sample, and values are expressed relative to the untreated control group set to 1.0

### Statistical analysis

All data are presented as mean ± SEM from at least three independent biological replicates (*n* = 3). Statistical analysis was performed in GraphPad Prism (v10) using one-way ANOVA followed by Tukey’s multiple-comparison post hoc test for group comparisons, unless stated otherwise. A *p* < 0.05 was considered statistically significant. Significance levels are denoted as *p < 0.05, **p < 0.01, ***p < 0.001, ****p < 0.0001.

## Supplementary information


Full_uncropped_blots


## Data Availability

All data generated or analyzed during this study are included in this published article.
