# Supplementary material for: Sustained dysregulation of iron and glutathione homeostasis induces chronoferroptosis, a persistent ferroptotic adaptation in neuronal cells
Source: Cell Death Discov. 2026 Jun 18;12:349. doi: 10.1038/s41420-026-03208-6 (PMC13522479; doi:10.1038/s41420-026-03208-6)

Figure 1 (C) FTH1 expression after acute and chronic Iron exposure in SH-SY5Y cells

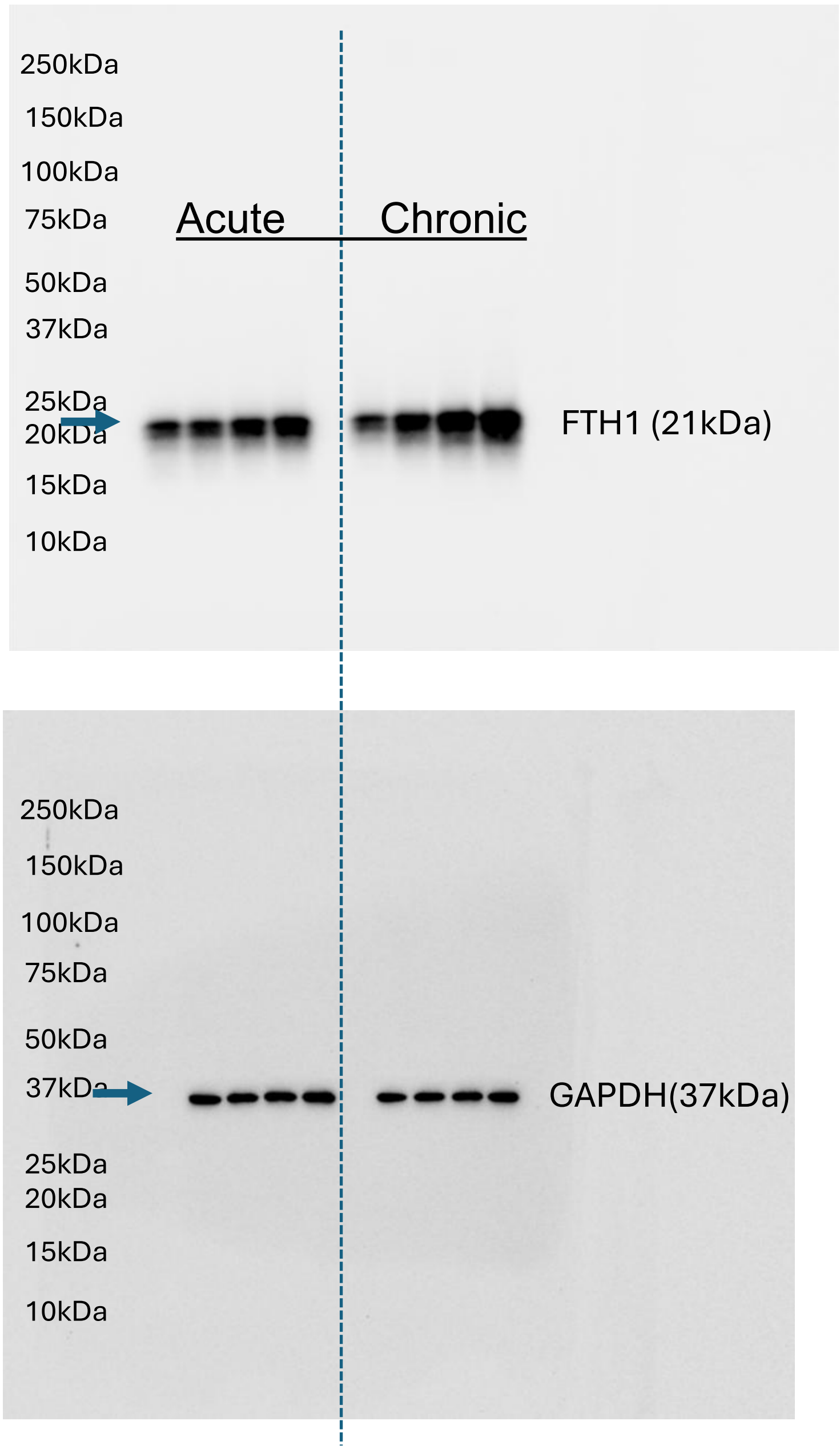

Figure in manuscript

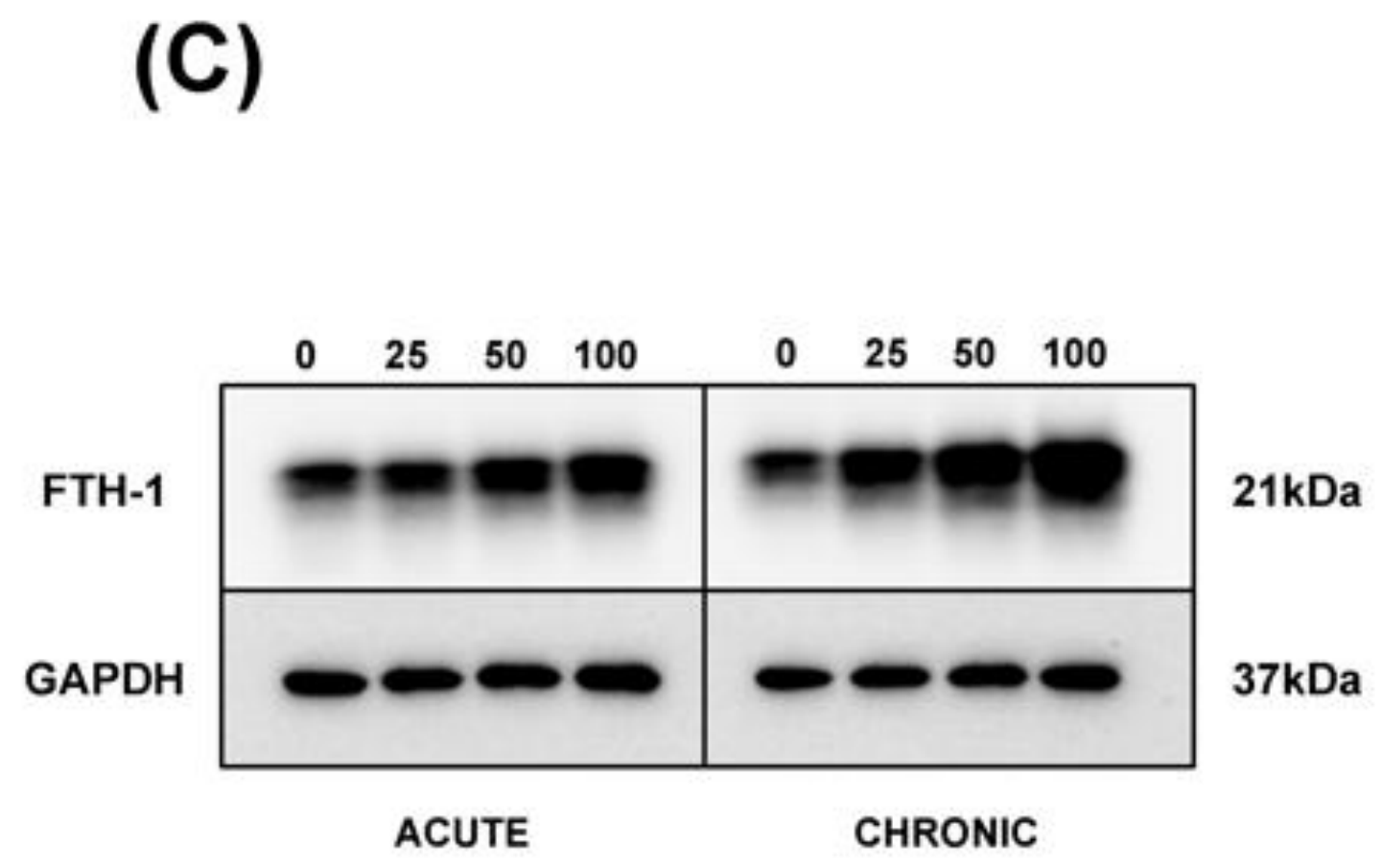

Figure 1 (D) FTH1 expression after acute and chronic BSO exposure in SH-SY5Y cells

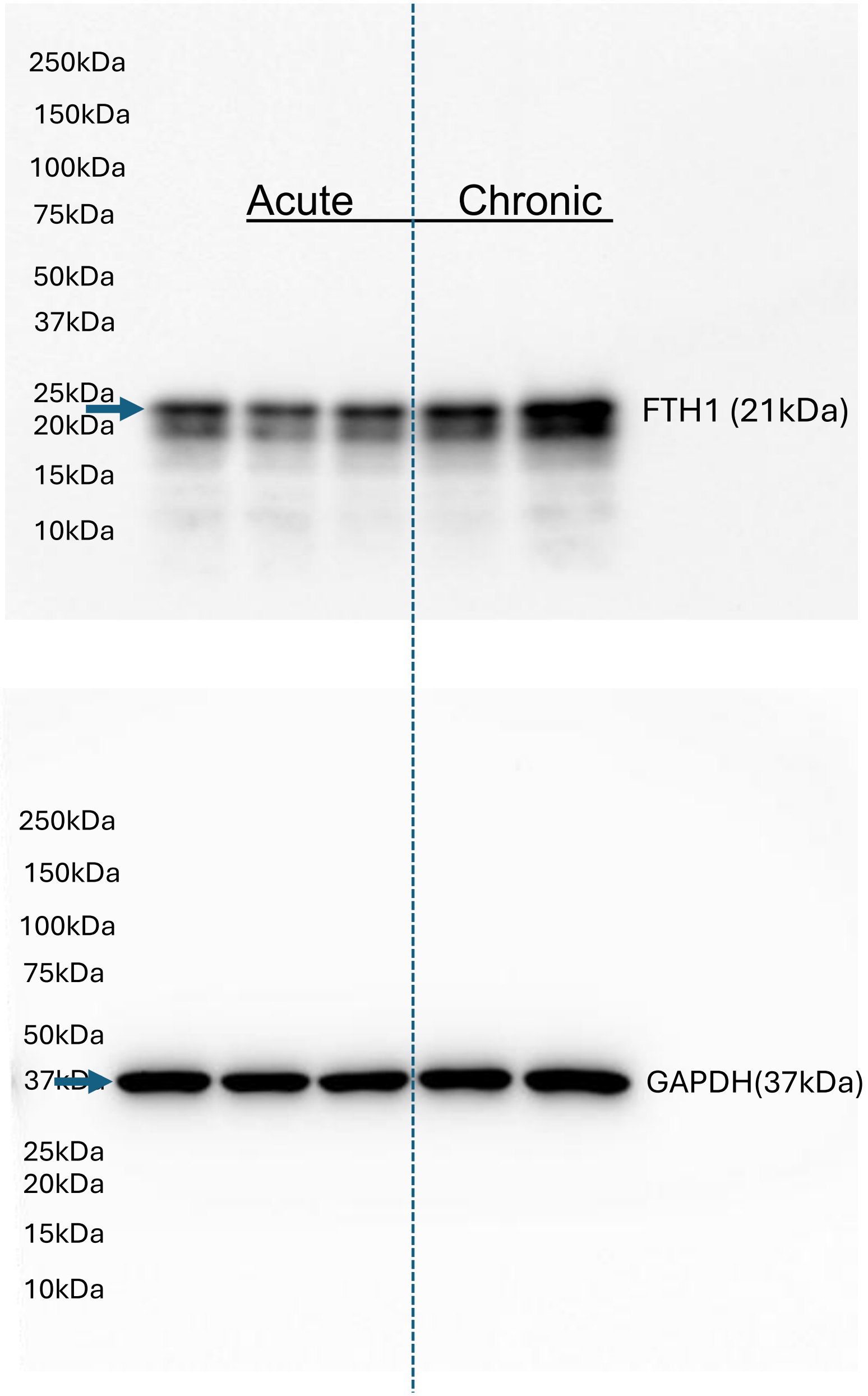

(D) Figure in manuscript

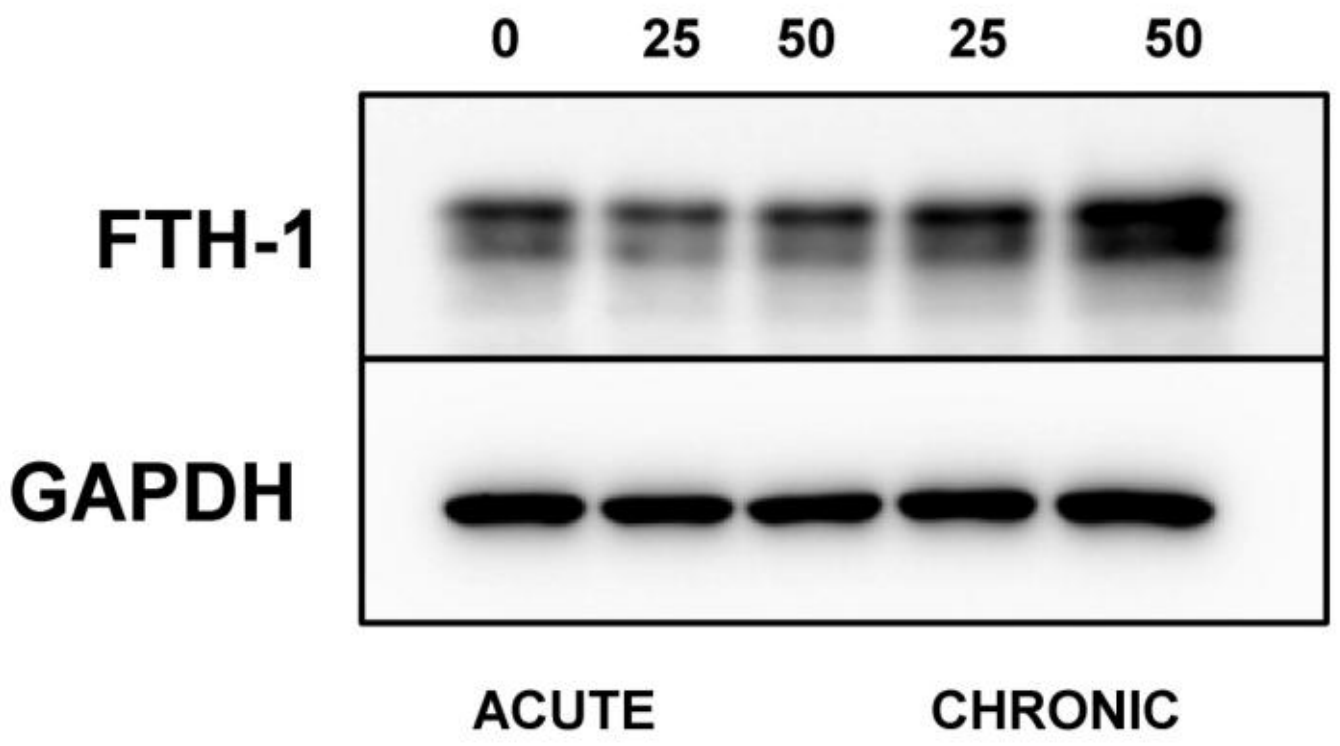

Figure 2 (A) GPX4 expression after acute and chronic Iron exposure in SH-SY5Y cells

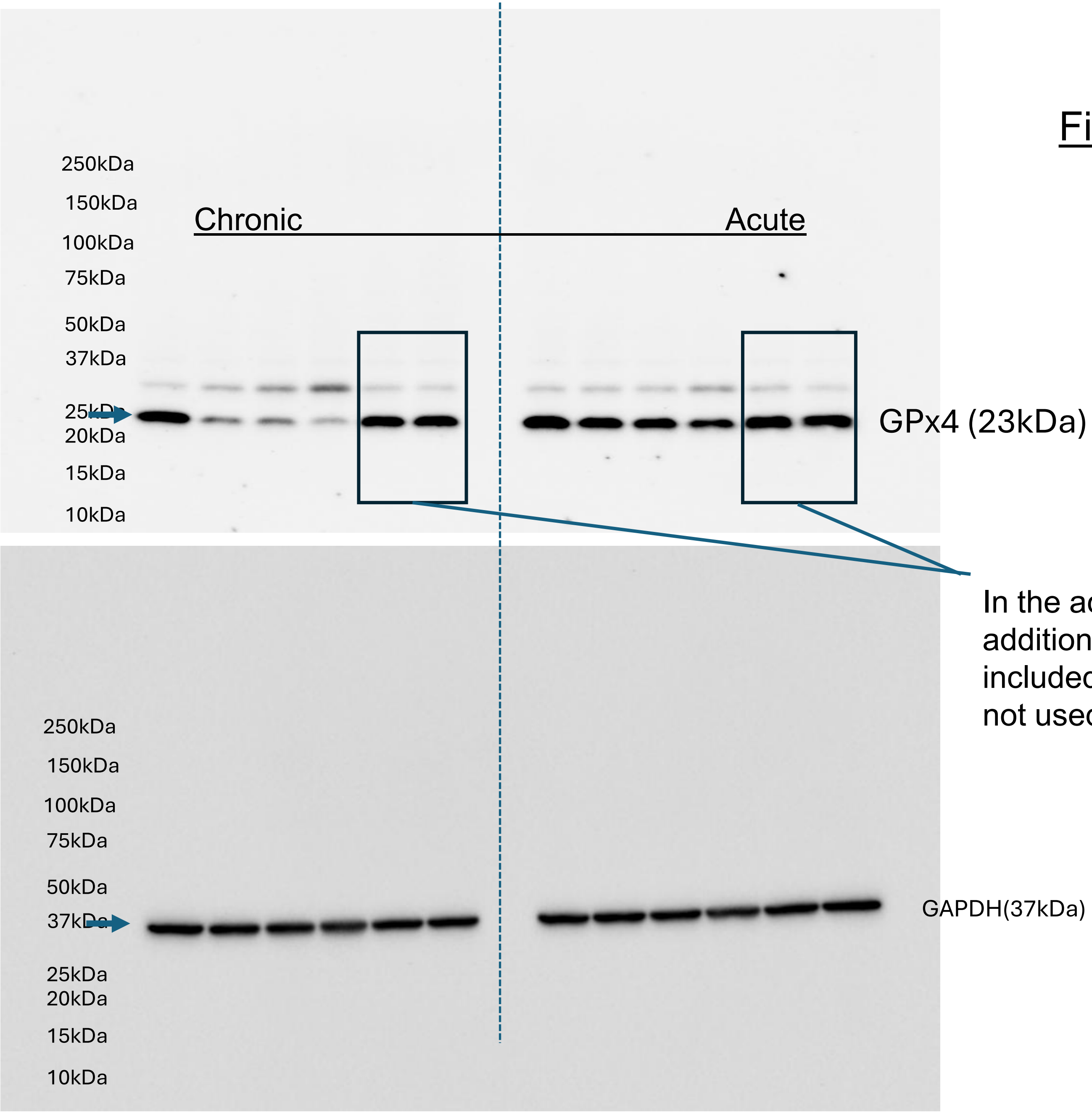

In the acute and chronic experiments, additional untreated control lanes were included on the same membrane but were not used for quantification

Figure in manuscript

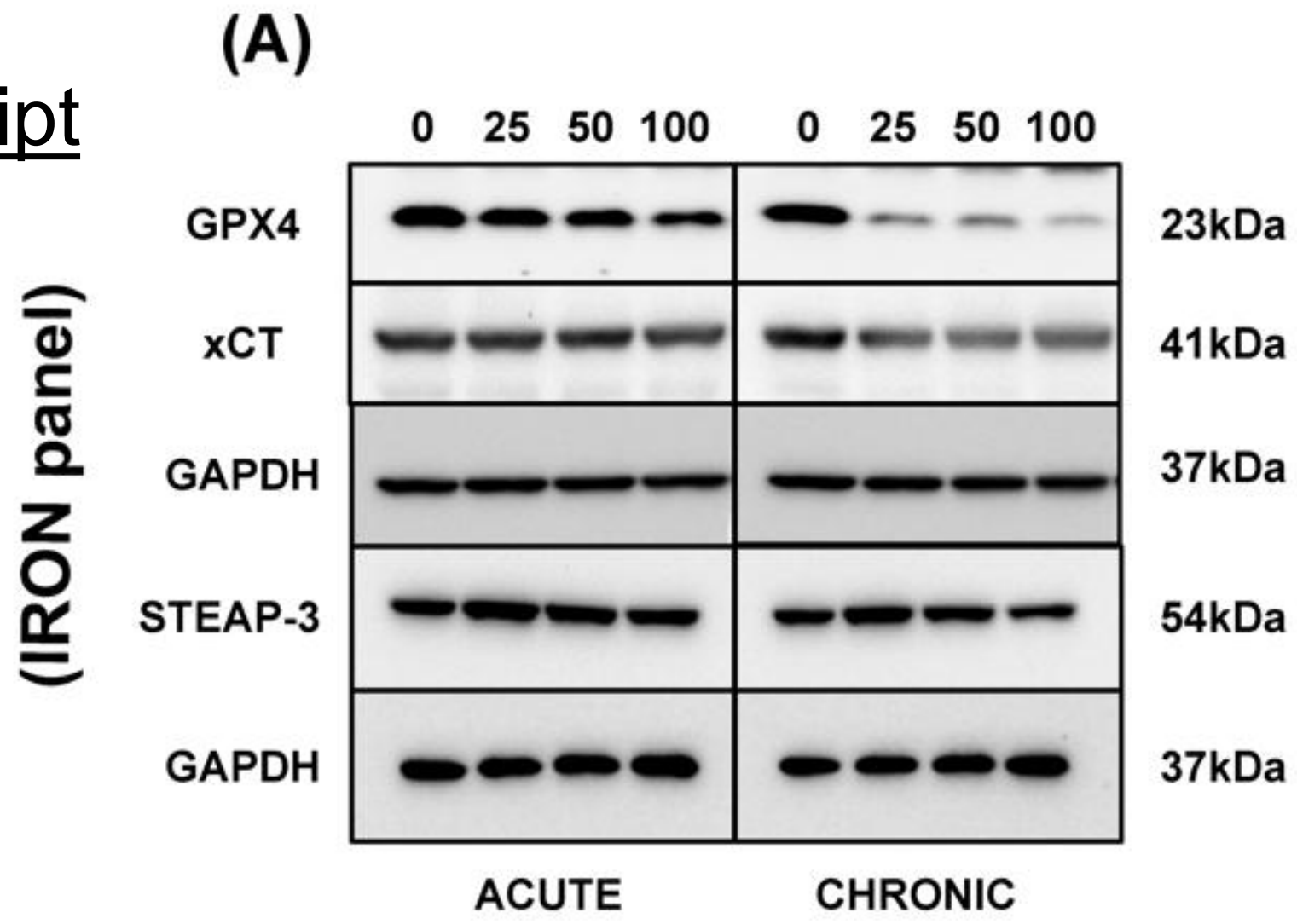

Figure 2 (A) xCT expression after acute and chronic Iron exposure in SH-SY5Y cells

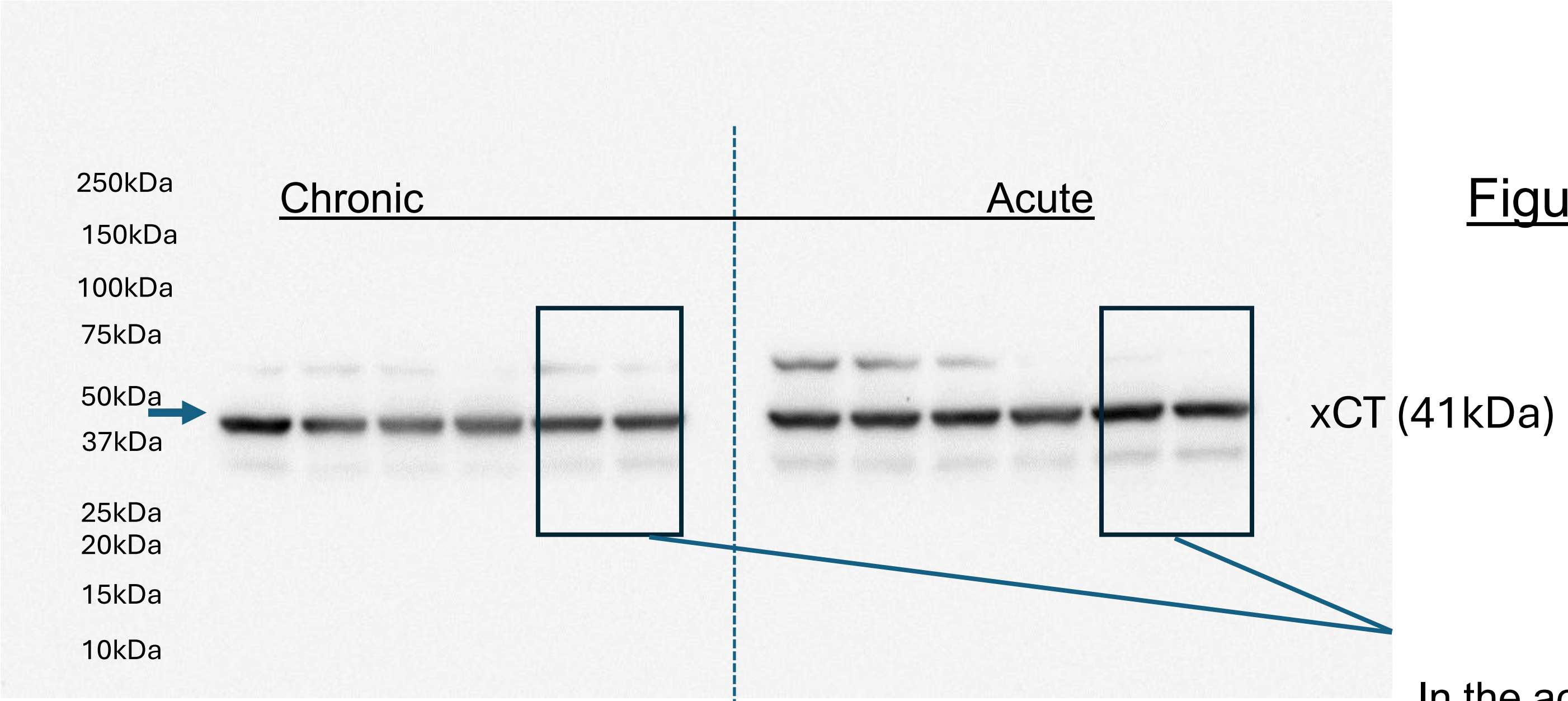

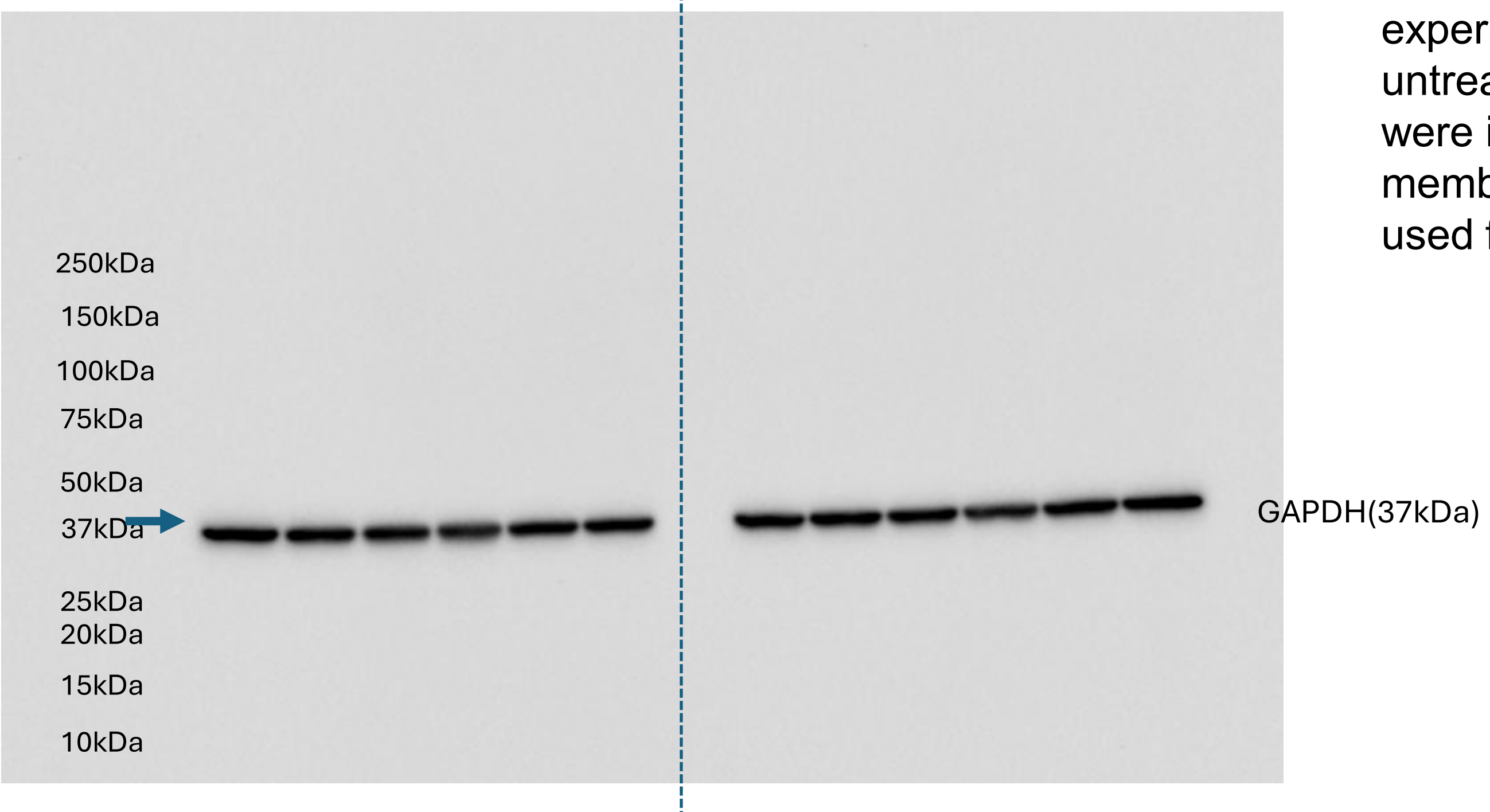

Figure in manuscript

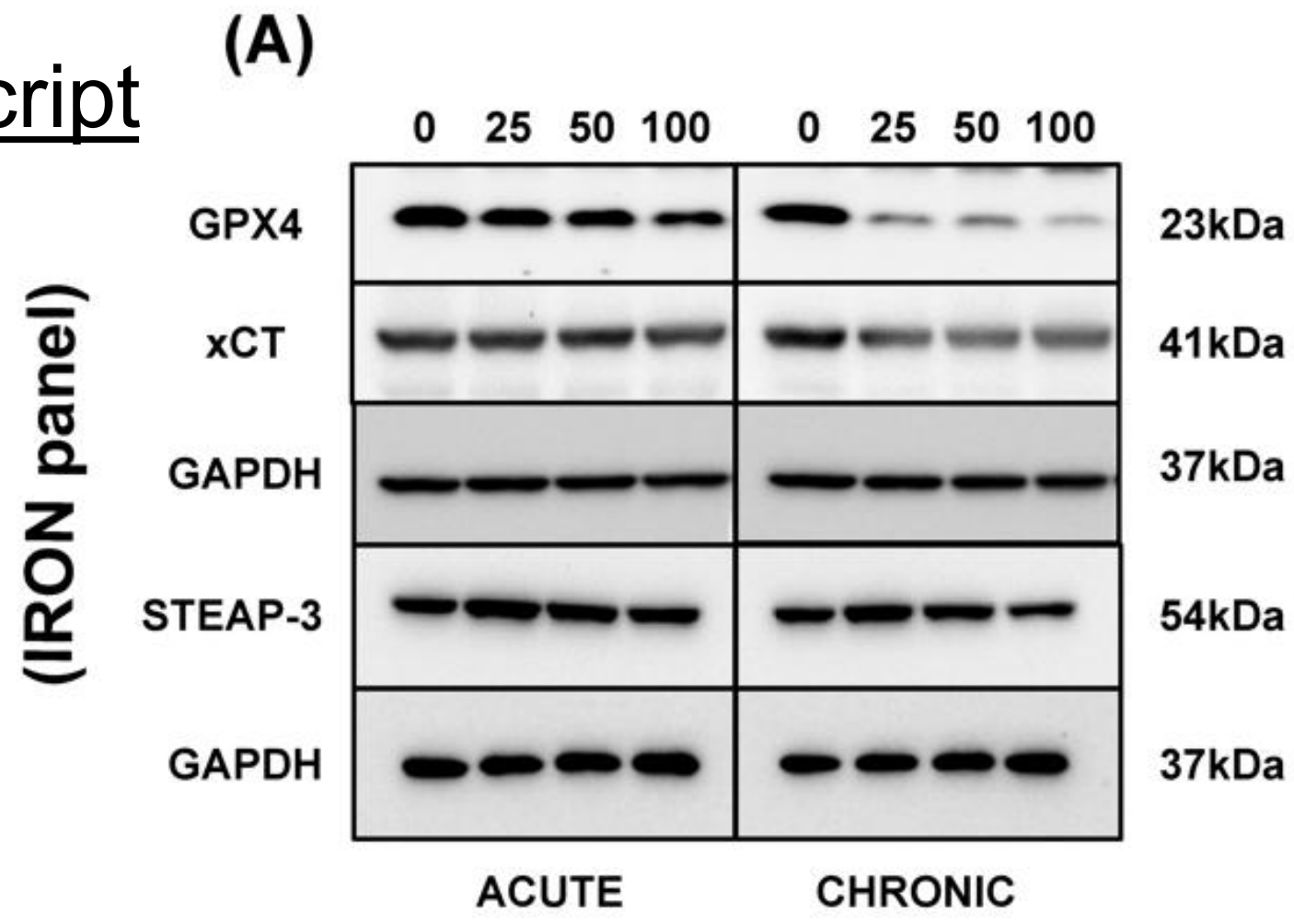

In the acute and chronic experiments, additional untreated control lanes were included on the same membrane but were not used for quantification

Figure 2 (A) STEAP-3 expression after acute and chronic Iron exposure in SH-SY5Y cells

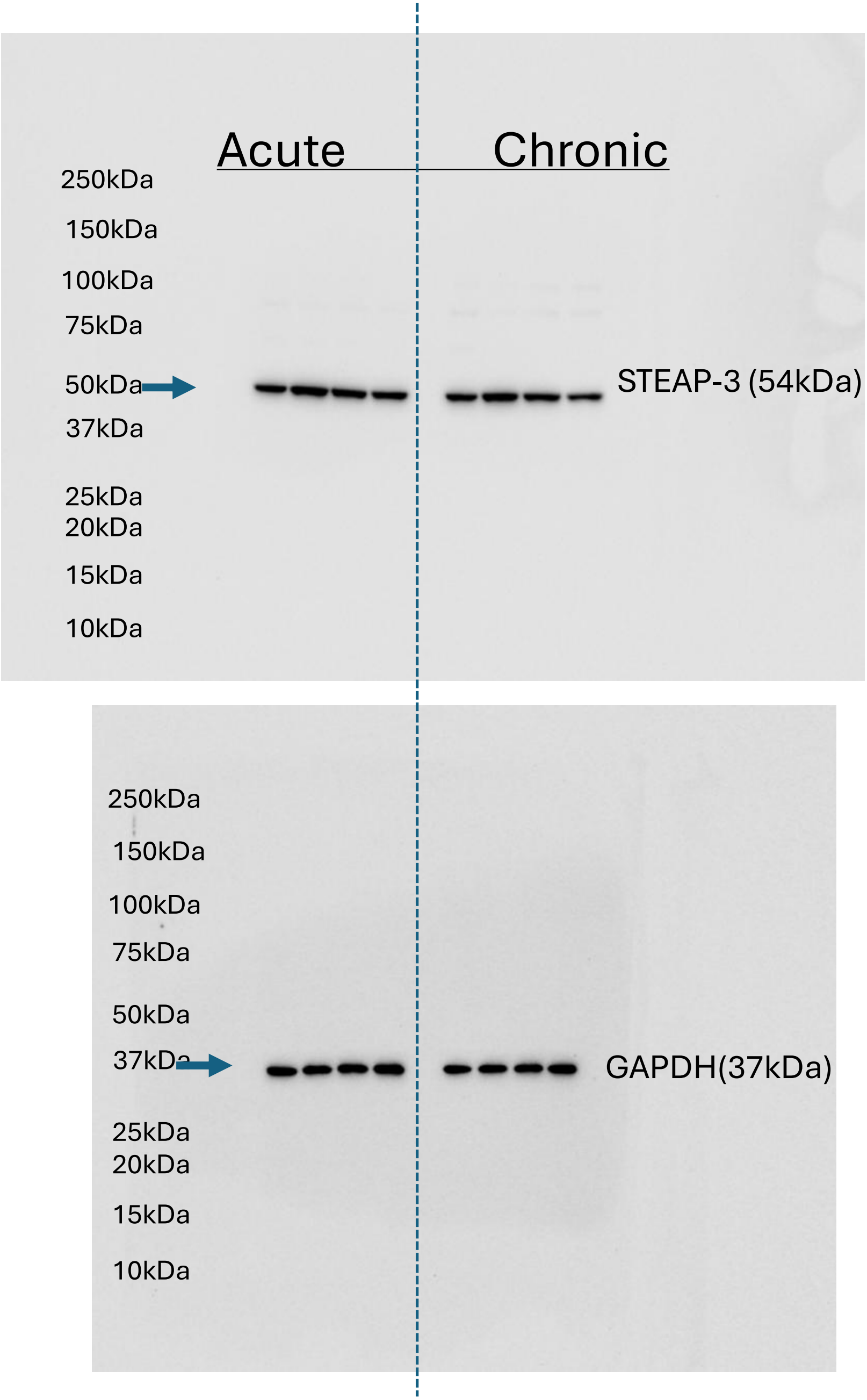

Figure in manuscript

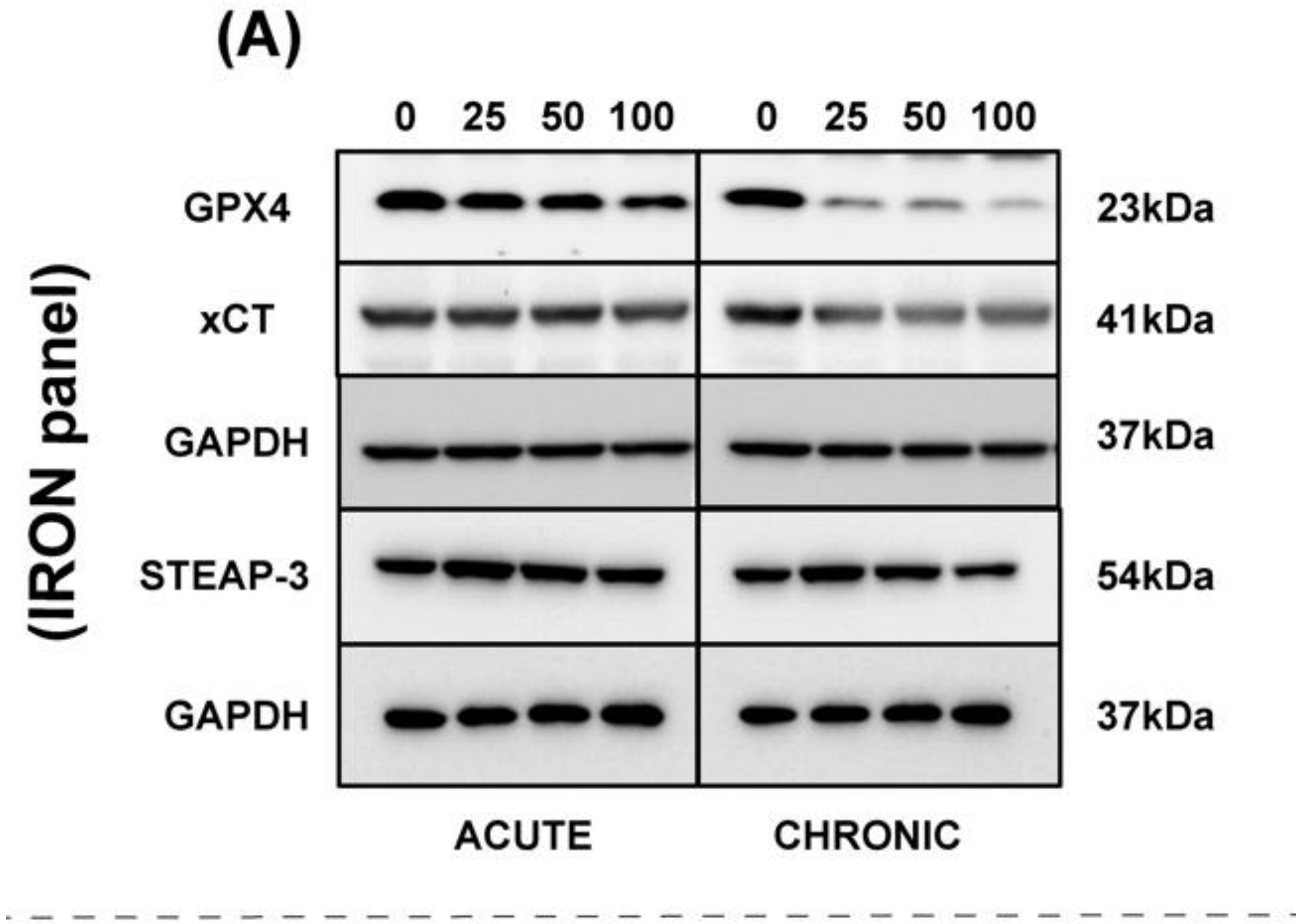

Figure 2 (E) GPX4 expression after acute and chronic BSO exposure in SH-SY5Y cells

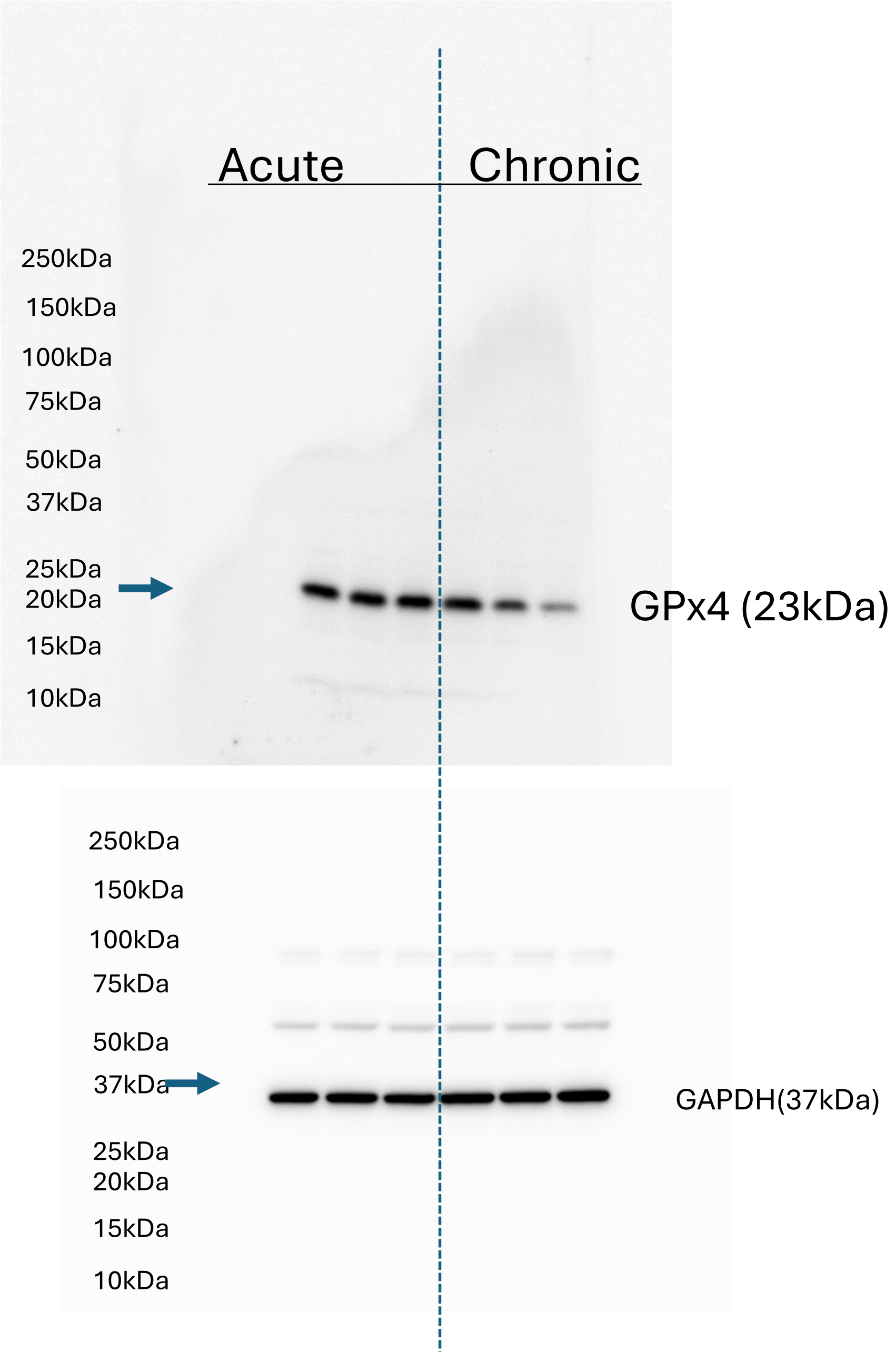

Figure in manuscript

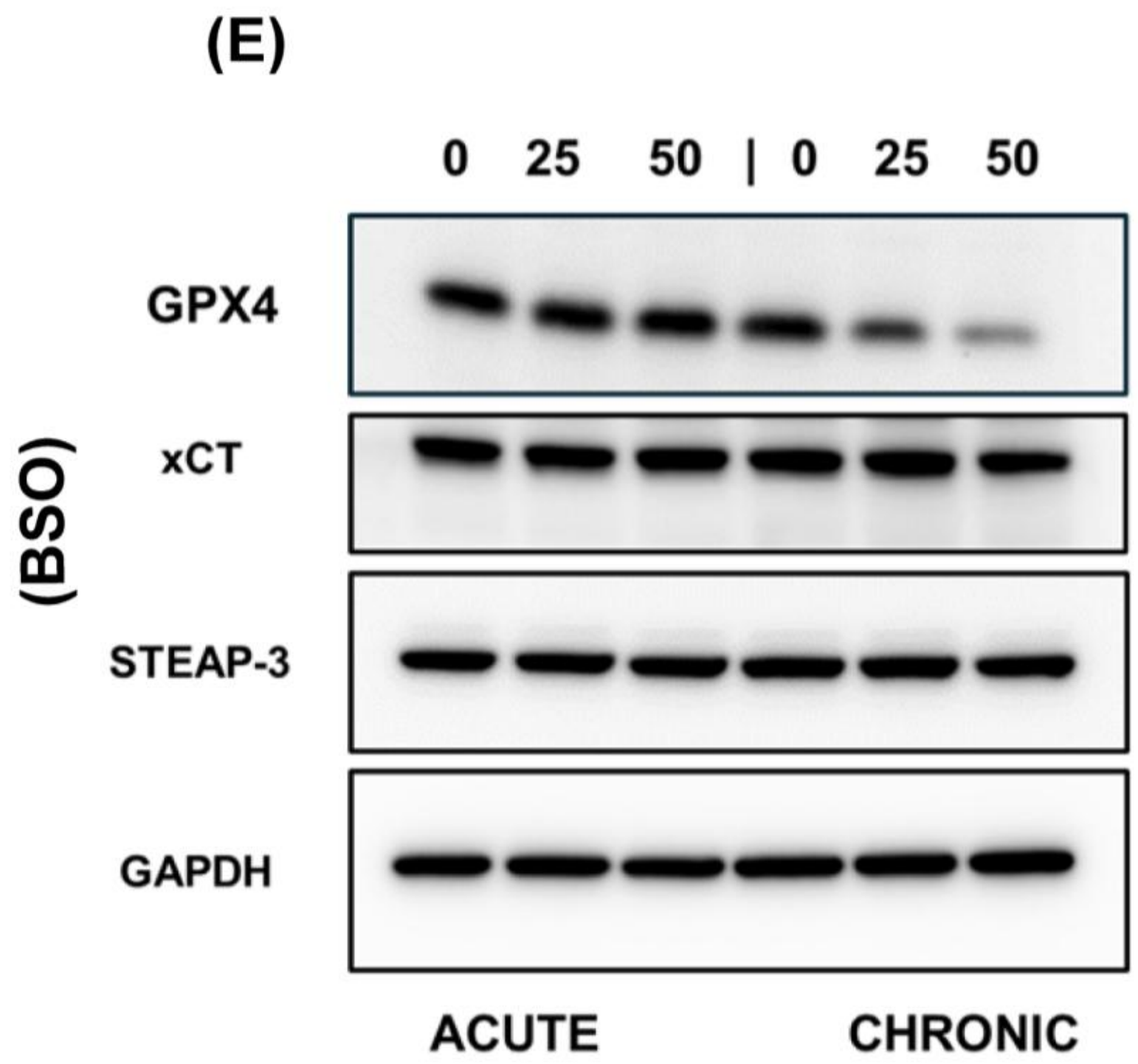

Figure 2 (E) xCT expression after acute and chronic BSO exposure in SH-SY5Y cells

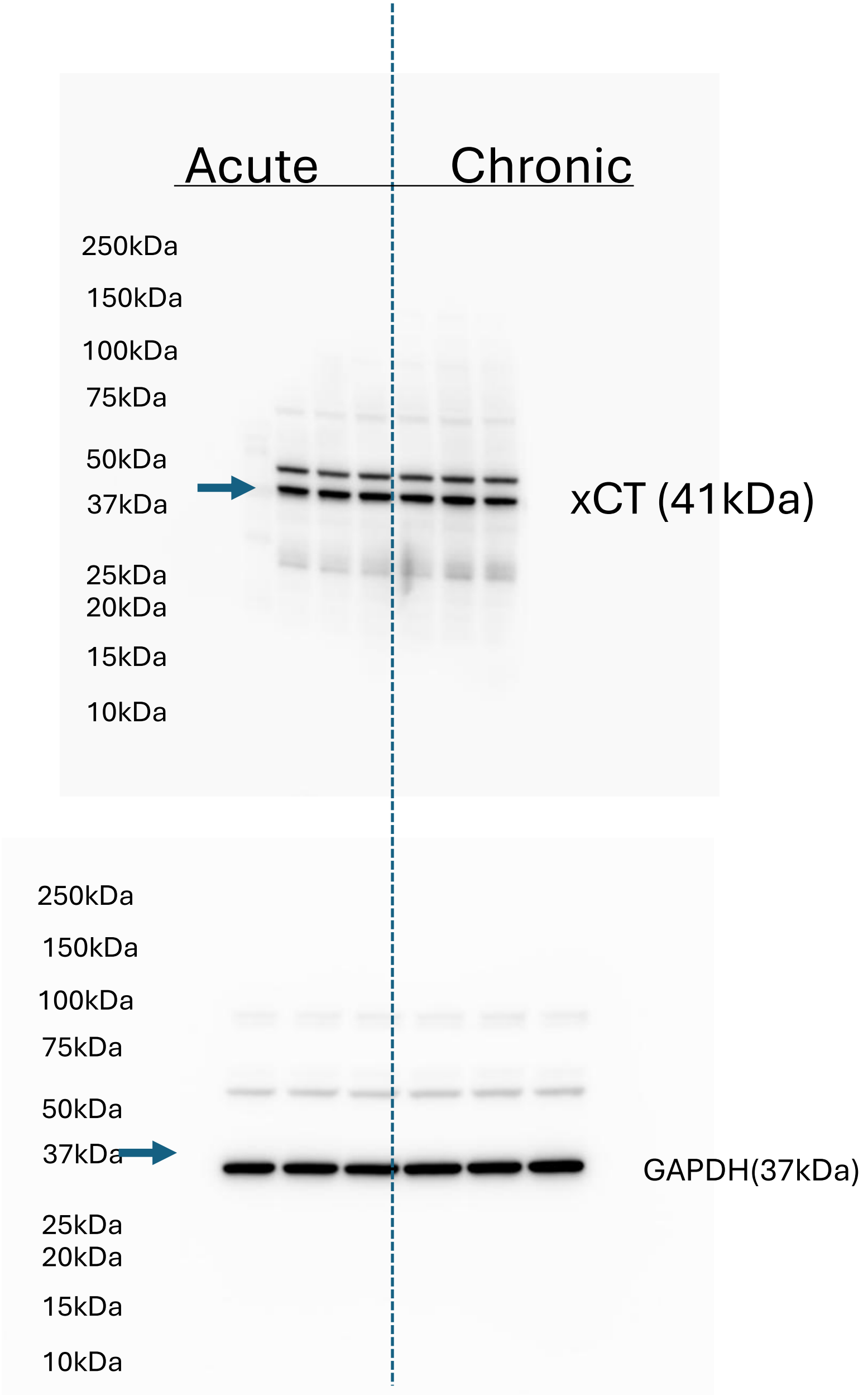

Figure in manuscript

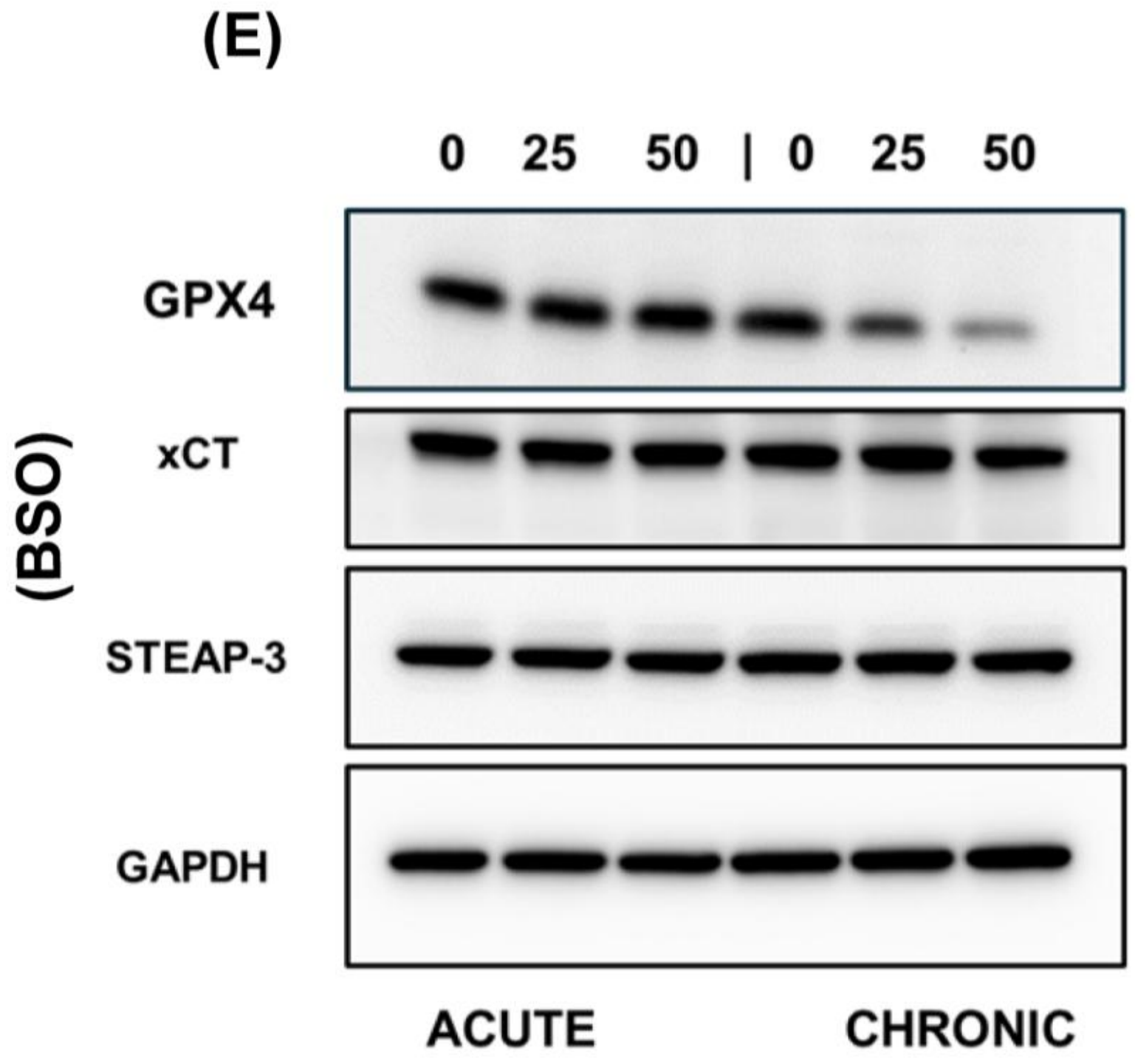

Figure 2 (E) STEAP-3 expression after acute and chronic BSO exposure in SH-SY5Y cells

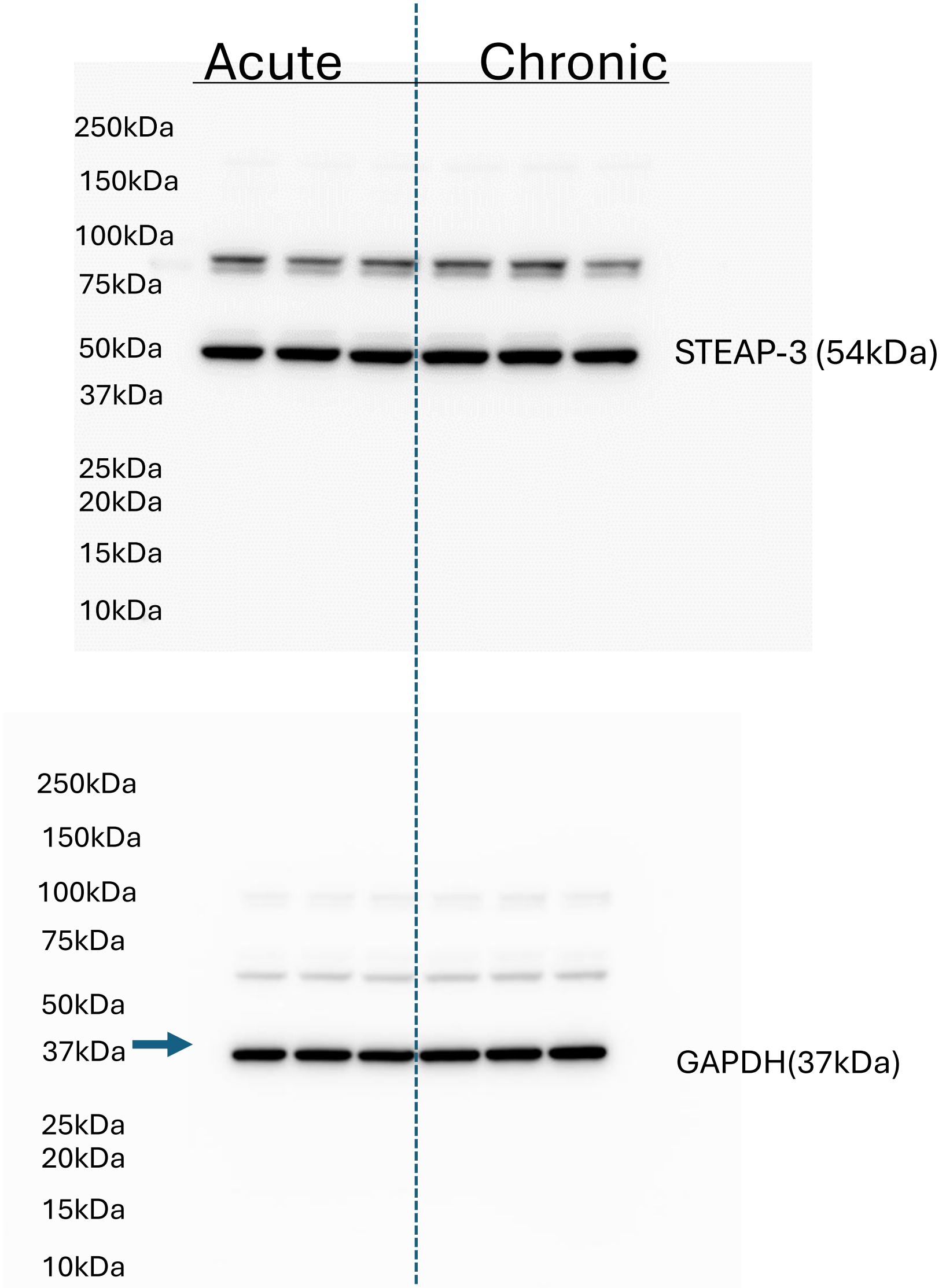

Figure in manuscript

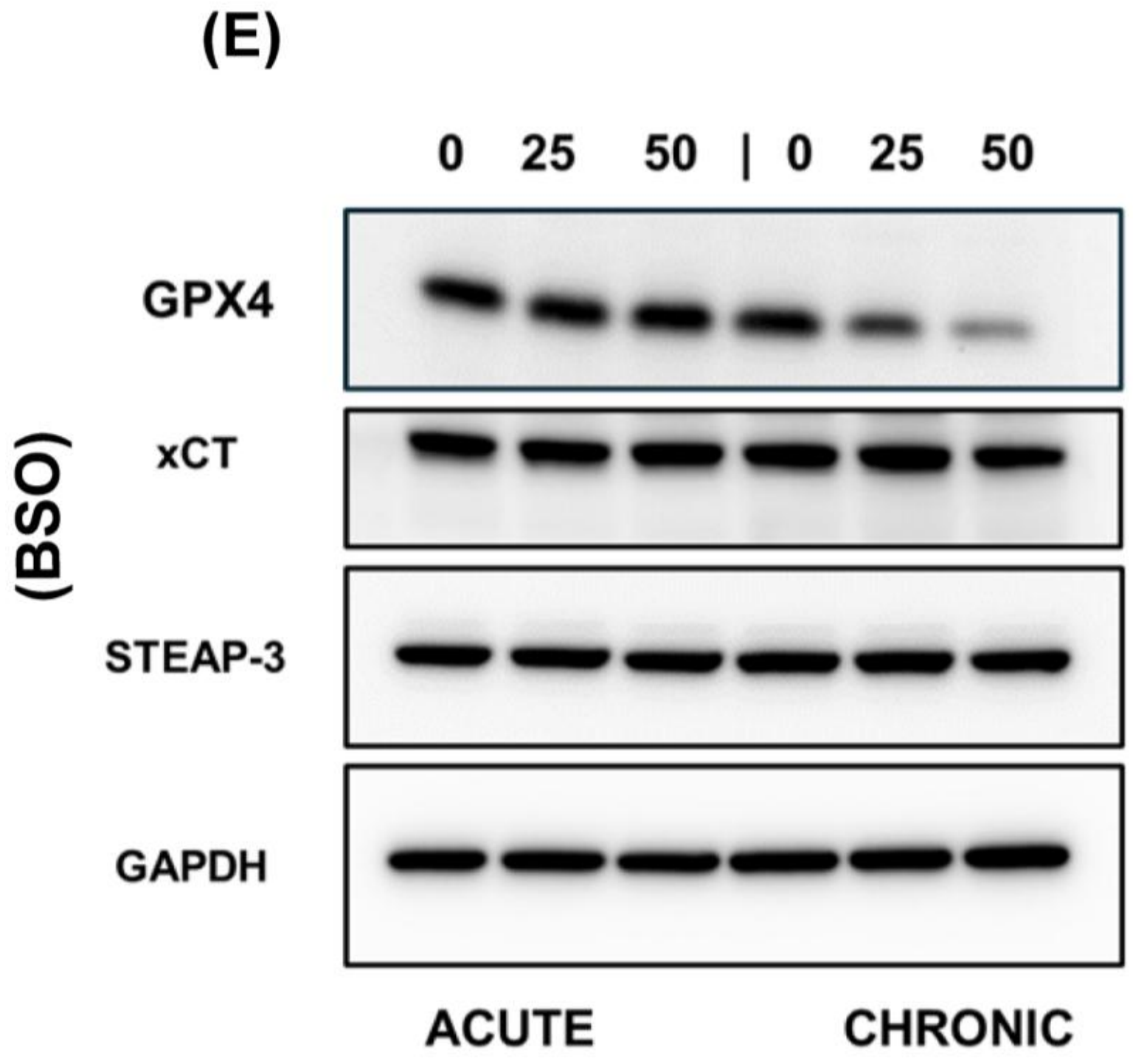

Supplement: Supplementary file 1 [file 41420_2026_3208_MOESM1_ESM.pdf]
